# Comparative methods for quantifying plasma biomarkers in Alzheimer's disease: Implications for the next frontier in cerebral amyloid angiopathy diagnostics

**DOI:** 10.1002/alz.13510

**Published:** 2023-10-31

**Authors:** Ryan T. Muir, Zahinoor Ismail, Sandra E. Black, Eric E. Smith

**Affiliations:** ^1^ Calgary Stroke Program Department of Clinical Neurosciences University of Calgary Calgary Alberta Canada; ^2^ Department of Community Health Sciences University of Calgary Calgary Alberta Canada; ^3^ Hotchkiss Brain Institute University of Calgary Calgary Alberta Canada; ^4^ Department of Psychiatry University of Calgary Calgary Alberta Canada; ^5^ Division of Neurology Department of Medicine Sunnybrook Health Sciences Centre Toronto Ontario Canada; ^6^ LC Campbell Cognitive Neurology Research Unit Dr Sandra Black Centre for Brain Resilience and Recovery, and Hurvitz Brain Sciences Program Sunnybrook Research Institute University of Toronto Toronto Ontario Canada

**Keywords:** Alzheimer's disease, amyloid, biomarker, cerebral amyloid angiopathy, cerebrospinal fluid, diagnostic test performance, intracerebral hemorrhage, plasma, tau

## Abstract

Plasma amyloid beta (Aβ) and tau are emerging as accessible biomarkers for Alzheimer's disease (AD). However, many assays exist with variable test performances, highlighting the need for a comparative assessment to identify the most valid assays for future use in AD and to apply to other settings in which the same biomarkers may be useful, namely, cerebral amyloid angiopathy (CAA). CAA is a progressive cerebrovascular disease characterized by deposition of Aβ_40_ and Aβ_42_ in cortical and leptomeningeal vessels. Novel immunotherapies for AD can induce amyloid‐related imaging abnormalities resembling CAA‐related inflammation. Few studies have evaluated plasma biomarkers in CAA. Identifying a CAA signature could facilitate diagnosis, prognosis, and a safer selection of patients with AD for emerging immunotherapies. This review evaluates studies that compare the diagnostic test performance of plasma biomarker techniques in AD and cerebrovascular and plasma biomarker profiles of CAA; it also discusses novel hypotheses and future avenues for plasma biomarker research in CAA.

## 1 INTRODUCTION

Alzheimer's disease (AD) is characterized by the accumulation of extracellular amyloid beta (Aβ) plaques and intracellular phosphorylated tau (p‐tau) tangles.^1^ Cerebral amyloid angiopathy (CAA) occurs when Aβ accumulates in the adventitia and media of small and medium vessels of the cerebral cortex, leptomeninges, and cerebellum.^2^, ^3^, ^4^, ^5^ CAA increases with age and occurs in up to 80% of those with AD; however, CAA can occur independent of AD with less than half of CAA cases meeting pathologic criteria for AD.^2^, ^3^ CAA is a major cause of microbleeds, hemorrhagic stroke, and vascular dementia—with intracerebral hemorrhage conferring great morbidity and mortality.^2^, ^3^, ^4^, ^5^

For decades, the diagnosis of AD has hinged on the characterization of the onset and progression of cognitive and behavioral symptoms, and resulting functional decline, in those with an amnestic profile of neurocognitive dysfunction. However, this diagnostic method is imperfect. Clinical diagnosis does not correlate well with pathologic diagnosis on *post mortem* assessment, which may be related to the clinical heterogeneity in cognitive presentation and overlap with other neurodegenerative and neurovascular conditions impairing other cognitive domains.^1^, ^6^ Over the past two decades, AD diagnostics have rapidly expanded to include neuroimaging biomarkers derived from amyloid and tau positron emission tomography (PET); cerebrospinal fluid (CSF) Aβ and tau analyses; and, more recently, plasma‐based biomarkers of p‐tau, total tau (t‐tau), and Aβ.^1^ The most validated CSF biomarkers in AD are a decreased Aβ_42/40_ ratio, as well as increased t‐tau and p‐tau181, with accumulating evidence for p‐tau231, and p‐tau217.^1^, ^7^, ^8^ Furthermore, in one study, CSF p‐tau/Aβ_42_ predicted the conversion from mild cognitive impairment (MCI) to AD dementia with 82.9% sensitivity and 90% specificity.^9^ However, the high cost and limited availability of PET, and the invasiveness of CSF collection, makes plasma biomarkers a more appealing approach for large‐scale screening efforts.^10^

**FIGURE 1 alz13510-fig-0001:**
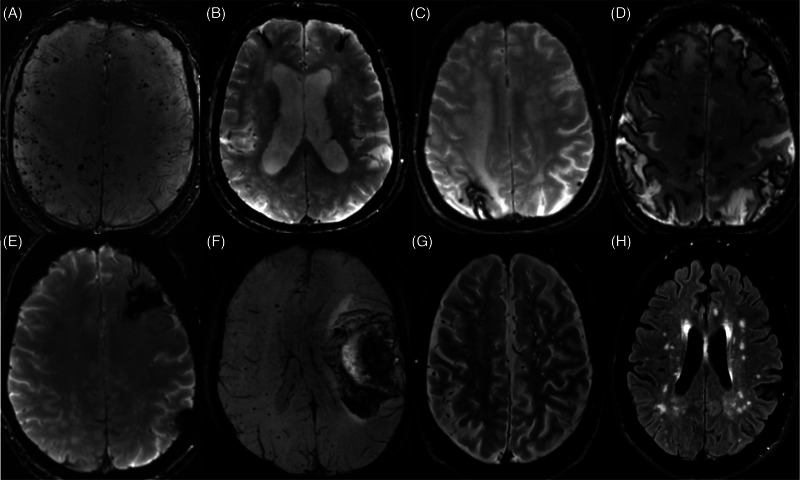
Neuroimaging characteristics of cerebral amyloid angiopathy. (A) SWI depicting multiple cortical cerebral microbleeds. (B) GRE depicting focal superficial siderosis in the bilateral frontal lobes. (C) GRE depicting focal superficial siderosis in the right posterior parietal lobe. (D) GRE depicting diffuse cortical superficial siderosis. (E) GRE depicting a focus of chronic hematoma cavity with hemosiderin staining in the left frontal lobe. (F) SWI depicting a large left frontal intracerebral hemorrhage with mild mass effect and midline shift. (G) T2‐weighted image demonstrating multiple enlarged perivascular spaces in the centrum semiovale of both cerebral hemispheres. (H) FLAIR sequence depicting white matter hyperintensities in a multi‐spot pattern throughout the centrum semiovale in bilateral hemispheres. FLAIR, fluid attenuated inversion recovery; GRE, T2*‐weighted gradient recalled echo; SWI, susceptibility weighted image.

As with AD, a definitive diagnosis of CAA is only possible with neuropathology. Efforts to identify neuroimaging biomarkers of CAA have improved diagnostic accuracy.^2^, ^3^, ^4^, ^5^ Neuroimaging biomarkers validated in prior iterations of the Boston criteria for CAA include cortical superficial siderosis, convexity subarachnoid hemorrhage, microbleeds, and intracerebral lobar hemorrhage.^2^, ^3^, ^4^, ^5^ The 2022 Boston criteria 2.0 for CAA now also include supportive criteria of enlarged perivascular spaces in the centrum semi‐ovale and white‐matter hyperintensities in a multi‐spot pattern—the non‐hemorrhagic neuroimaging features of CAA.^2^ However, the diagnostic specificity remains limited. The current Boston criteria 2.0, for distinguishing probable compared to no CAA, have a sensitivity of 80% (95% confidence interval [CI]: 71%–88%) and specificity of 82% (95% CI: 62%–94%), though when distinguishing probable or possible compared to no CAA these criteria have a sensitivity of 90% (CI: 82%–95%) and specificity of 59% (95% CI: 39%–78%).^2^ The typical neuroimaging features of CAA on magnetic resonance imaging (MRI) according to Boston criteria 2.0 are depicted in Figure 1.

**RESEARCH IN CONTEXT**

1. **Systematic review**: Pubmed/MEDLINE searches identified studies which (a) compared diagnostic test performances of plasma amyloid beta (Aβ) and tau assays in Alzheimer's disease (AD), and (b) characterized spinal fluid and plasma biomarkers of cerebral amyloid angiopathy (CAA).
2. **Interpretation**: A comparative assessment of plasma biomarker assay performance identified several plasma biomarker assays with variable test performance in AD. While mass spectrometry displayed the best diagnostic test performance for plasma Aβ quantification, good to excellent performance was noted across most assays for tau. Furthermore, in CAA, few have evaluated spinal fluid biomarkers and there is a scarcity of evidence evaluating plasma biomarkers.
3. **Future directions**: This review identifies a need to characterize and validate CAA plasma biomarkers. Such markers could increase the feasibility and accuracy of CAA diagnosis in clinical practice, and potentially improve the prediction of risk for CAA‐related amyloid‐related imaging abnormalities in patients undergoing immunotherapies for AD. We propose novel hypotheses including a stage‐dependent alteration in plasma Aβ_40_ in CAA and potential implications for increasing the diagnostic specificity of the Boston criteria and informing hemorrhage risk.

Plasma biomarkers could have a role in CAA diagnosis. Enlarged perivascular spaces may reflect impaired glymphatic clearance across the neurovascular unit.^11^ Impaired perivascular drainage of Aβ is hypothesized to have a major role in the pathogenesis of CAA.^3^, ^12^ As CAA is a disorder of cerebral small vessels and results from the aberrant clearance and subsequent accumulation of amyloid surrounding and within small vessels, it is biologically plausible that measuring the spillage of these poorly cleared waste products into plasma, across the neurovascular unit, may serve as a useful approach to directly investigate and diagnose CAA. Furthermore, given the proposed selective dysfunction in the neurovascular glymphatic interface in CAA, we might expect a different plasma biomarker profile in CAA compared to AD without CAA. Identifying plasma biomarkers of CAA could facilitate accurate and early diagnosis, which may be the next natural advancement to further increase the diagnostic sensitivity and specificity of the Boston criteria. However, CSF and plasma amyloid have been relatively less studied in CAA compared to AD.[Fig alz13510-fig-0001]

**FIGURE 2:**
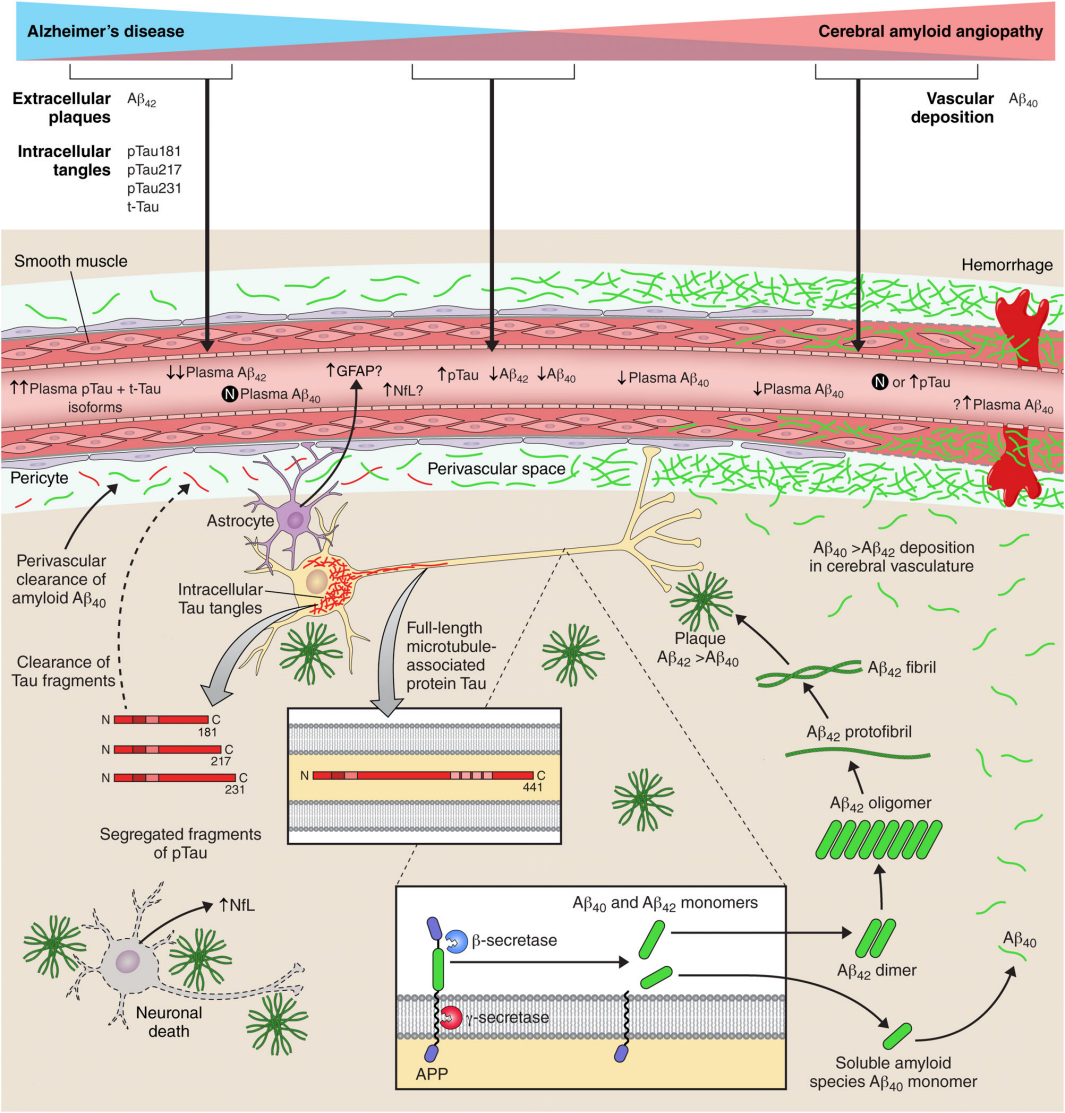
Pathophysiologic spectrum of Alzheimer's disease and cerebral amyloid angiopathy and hypothesized plasma biomarker alterations across the spectrum. APP is metabolized by β‐secretase and γ‐secretase to produce Aβ isoforms between 27 and 43 amino acids in length.^1^, ^14^ When APP is metabolized abnormally two pathologic isoforms are generated: Aβ_40_ and Aβ_42_. Aβ _42_ is the least soluble, and has a higher propensity to aggregate and deposit in the brain parenchyma as neuritic plaques in AD.^1^, ^3^, ^13^ The shorter Aβ_40_ isoform is more soluble and is preferentially cleared along perivascular pathways.^1^, ^3^, ^13^ In some persons, beta‐amyloid deposits within small cerebral arteries and arterioles to cause CAA.^2^, ^12^, ^14^. The deposition of beta‐amyloid weakens cerebral vessels, resulting in loss of smooth muscle and subsequent hemorrhaging. While Aβ_42_ primarily deposits in plaques, it also deposits within cortical and leptomeningeal small vessels in CAA, but the ratio of Aβ_40_ : Aβ_42_ is higher in cerebral blood vessels than in the parenchymal amyloid plaques.^3^ Furthermore, in AD, intracellular tau tangles also accumulate and are responsible for neuronal injury. Tau tangles of various lengths (p‐tau181, p‐tau217, p‐tau231) are elevated in AD and can also be measured in plasma. It is less understood what corresponding changes in p‐tau fragments, if any, occur in pure CAA. Another potential contributor to neuronal injury in AD is reactive astrocytosis, with corresponding elevations in plasma GFAP demonstrated in some studies. Furthermore, neurofilaments are liberated from injured/apoptotic neurons and plasma NfL has been demonstrated to be elevated AD, but likely is a non‐specific elevation arising as a consequence of axonal injury. Aβ, amyloid beta; AD, Alzheimer's disease; APP, amyloid precursor protein; CAA, cerebral amyloid angiopathy; GFAP, glial fibrillary acidic protein; NfL, neurofilament light chain; p‐tau, phosphorylated tau.

The pathophysiologic spectrum of AD and CAA, their overlap, and summary of amyloid and tau synthesis are summarized in Figure 2 along with hypothesized corresponding changes in plasma biomarkers across this disease spectrum. The amyloidogenic pathway through β‐ and γ‐ secretases generate aberrant Aβ isoforms. Aβ_42_ is the least soluble, and has a higher propensity to aggregate and deposit as parenchymal plaques in AD.^1^, ^3^, ^13^ The shorter Aβ_40_ isoform is more soluble and is preferentially cleared along perivascular pathways.^1^, ^3^, ^13^ In those with CAA, Aβ_40_ deposits within and causes damage to cerebral small blood vessels.^2^, ^12^, ^14^ While the traditional understanding of the pathobiology of AD has centered on injury mediated by amyloid plaque and tau tangles, there is increasing appreciation for the pathophysiologic role of a reactive astrocytosis as a pathophysiologic pathway interacting with amyloid pathology.^15^, ^16^ One potential biomarker of reactive astrocytosis is plasma glial fibrillary acidic protein (GFAP), which is elevated in AD and is a marker of early cerebral amyloidosis, but not tauopathy.^15^, ^16^ While few studies have examined GFAP in CAA, a recent study found no association between GFAP and CAA pathology.^16^ Additionally, neurofilaments are cytoskeletal scaffolding components inside neurons that may serve as sensitive markers of neuronal injury.^17^, ^18^ Neurofilament light chain (NfL) is a marker of axonal injury and is elevated in AD and across several neurodegenerative disorders of the central nervous system.^17^, ^18^

The recent rapid growth in plasma biomarker studies in AD has been enabled by the development of accessible, reliable, and more accurate techniques. Historically, enzyme linked immunosorbent assay (ELISA) methodologies were implemented, which have limited capacity to detect biomarkers in the small quantities present in plasma.^19^ With the advent of single molecule protein detection (Simoa) and immunoprecipitation mass spectrometry (IP‐MS) assays, the ability to detect these biomarkers in plasma has improved. In AD, plasma Aβ_42_ is decreased, while t‐tau, p‐tau181, p‐tau231, p‐tau217, GFAP, and neurofilaments (NfL) are elevated.^20^, ^21^, ^22^, ^23^, ^24^ The plasma Aβ_42 /40_ ratio, p‐tau181, p‐tau217, p‐tau231, and t‐tau have emerged as potentially sensitive and specific measures to discriminate AD.^20^, ^21^, ^25^, ^26^, ^27^, ^28^, ^29^, ^30^, ^31^, ^32^

As exciting as this rapidly expanding plasma biomarker space is, a critical review of the various methodologies now available is warranted to identify the biomarkers and techniques with the best accuracy and reliability for future consideration. The first objective of this narrative review is to provide a focused overview of recent studies that have directly compared the diagnostic performance of different assays used to quantify plasma biomarkers in AD in reference to standard diagnostic tests (PET, CSF biomarkers, or pathology) or in reference to conversion to clinical AD dementia or compared to healthy cognitively normal controls. Identifying studies that have directly compared methods within the same group of participants and using the same diagnostic reference standard for those participants facilitates a more homogenous and accurate comparison of plasma diagnostic test performance characteristics, as opposed to heterogenous comparisons of different methods in different participants across different studies. The results of this first objective will identify the most valid biomarker assays for future use in AD and identify valid assays to then be applied to future CAA research. Building on our first objective, our second aim is to review CSF and plasma biomarker studies that have been published in CAA to identify knowledge gaps and opportunities to apply the most valid assays identified from our first objective to CAA. Furthermore, we will discuss the diagnostic and potential treatment implications of these findings for AD and CAA.

## 2 LITERATURE REVIEW

In accordance with methodological standards for narrative reviews, we followed the Scale for the Assessment of Narrative Review Articles (SANRA; see Table S1 in supporting information).^33^

MEDLINE/PubMed was searched between January 1, 2016 and November 6, 2022. Search terms included: (serum or plasma) AND biomarker AND (tau or p‐tau or t‐tau or Aβ or amyloid or amyloid beta or NfL or neurofilament or GFAP or glial fibrillary acidic protein) AND (MCI or mild cognitive impairment or Alzheimer's or Alzheimer or “cerebral amyloid angiopathy” or CAA or “amyloid angiopathy”) AND (sensitivity or specificity or diagnosis or diagnostic). All titles and abstracts were reviewed. Full text review was conducted for studies that examined any one of the following plasma biomarkers in participants at increased risk of AD, with MCI, AD or CAA: Aβ_40_, Aβ_42_, Aβ_40/42_ ratio, total tau, phosphorylated‐tau (p‐tau181, p‐tau217, p‐tau231), neurofilament (NfL), or GFAP.

To be included in this narrative review for our first objective, full‐text articles had to: (1) evaluate the area under the receiver operating curve (AUROC) of any one of the aforementioned plasma biomarkers in relation to a reference standard (CSF Aβ_42_, Aβ_40_, Aβ_42/40_; amyloid and/or tau PET; or neuropathology), conversion to AD dementia or compared to healthy controls (HC); and (2) directly compare the diagnostic test performance of two or more different plasma biomarker quantification techniques. We extracted area under the curve (AUC) reported by studies to three decimal places and corresponding 95% CIs where available. In some studies, AUCs are reported to two decimal places, so in these instances we have reported AUCs to two decimal places.

For our second objective, MEDLINE/PubMed was searched from inception to November 6, 2022 using: (serum or plasma or CSF or cerebrospinal fluid) AND (tau or p‐tau or t‐tau or Aβ or amyloid or amyloid beta) AND (“cerebral amyloid angiopathy” or CAA or “amyloid angiopathy”). Articles evaluating either plasma or CSF tau or Aβ in CAA were reviewed in full and included in our Results section. We included articles that diagnosed CAA in accordance with Boston criteria or pathology or genetic CAA.

## 3 RESULTS

For our literature search pertaining to our main objective, 1185 publications were identified for title and abstract review. Of these, 83 articles were identified for full text review, and 9 met inclusion criteria directly comparing the AUROCs of different laboratory techniques used to quantify plasma (1) Aβ and/or (2) tau.^34^, ^35^, ^36^, ^37^, ^38^, ^39^, ^40^, ^41^, ^42^ An inclusion flow diagram of the literature search is depicted in Figure S1 in supporting information. The five studies that examined and compared plasma Aβ methodologies are summarized in Table 1,^34^, ^35^, ^36^, ^37^, ^38^ while five studies that examined t‐tau and p‐tau plasma methodologies are summarized in Table 2.^38^, ^39^, ^40^, ^41^, ^42^

**TABLE 1:** Summary of studies directly comparing plasma Aβ biomarker quantification techniques in AD.

Study	Patient cohort(s)	Disease setting	Cognitively normal controls	Amyloid biomarkers	Plasma biomarker methods compared	Reference standard	Reference standard AUC (95% CI)	
Janelidze et al. (2021).^34^	*bioFINDER* MCI, *n* = 104	*bioFINDER*: MCI	*bioFINDER*: *n* = 182	Aβ_42/40_	IP‐MS‐ Washington University (IP‐MS‐WashU) Elecys Immunoassay Roche (IA‐Elc) liquid chromatography MS Araclon (LC‐MS‐Arc) Immunoassay Euroimmun (IA‐EI) Simoa immunoassay Quanterix (IA‐N4PE). IP‐MS–Shimadzu (IP‐MS‐Shim) IP‐MS–University of Gothenburg (IP‐MS‐UGOT) Simoa immunoassay Quanterix (IA‐Quan)	*bioFINDER*: F18 flutemetamol Amyloid PET CSF Aβ_42_/Aβ_40_	CSF Aβ_42_/Aβ_40_ *bioFINDER* IP‐MS‐WashU: 0.855 (0.810–0.899) IA‐Elc: 0.778 (0.725–0.832) LC‐MS‐Arc: 0.776 (0.721–0.830) IA‐EI: 0.697 (0.635–0.758) IA‐N4PE: 0.687 (0.626–0.748)	PET *bioFINDER* IP‐MS‐WashU: 0.833 (0.787–0.879) IA‐Elc: 0.727 (0.669–0.784) LC‐MS‐Arc: 0.753 (0.696–0.811) IA‐EI: 0.672 (0.609–0.735) IA‐N4PE: 0.655 (0.591–0.719)
	*ADNI* MCI, *n* = 51 AD, *n* = 20	*ADNI*: MCI + AD	*ADNI*: *n* = 51			*ADNI*: F18 flutemetamol amyloid PET	*ADNI* N/A	*ADNI* IP‐MS‐WashU: 0.845 (0.772–0.917) IP‐MS‐Shim: 0.821 (0.747–0.895) IA‐Elc: 0.740 (0.651–0.829) IA‐N4PE: 0.685(0.590–0.781) IP‐MS‐UGOT: 0.662 (0.565–0.758) IA‐Quan: 0.634 (0.534–0.734)
	*Subcohorts*: (a)Shimadzu, Kyoto, Japan (b) University of Gothenburg and Simoa Quanterix	*Sub cohorts*: (a) Shimadzu, Kyoto, Japan Amyloid PET+, *n* = 86 (b) University of Gothenburg and Simoa Quanterix Amyloid PET+, *n* = 86	*Subcohorts*: (a) Shimadzu, Kyoto, Japan Amyloid PET–, *n* = 114 (b) University of Gothenburg and Simoa Quanterix Amyloid PET–, *n* = 141			*Subcohorts*: In this subcohort, CSF and PET were completely concordant in identifying amyloid positive patients;' therefore, AUCs for CSF and PET were the same F^18^ flutemetamol amyloid PET CSF Aβ_42_/Aβ_40_	*Shimadzu*: IP‐MS‐WashU: 0.872 (0.824–0.920) IP‐MS‐Shim: 0.825 (0.767–0.882) LC‐MS‐Arc: 0.775 (0.711–0.839) IA‐Elc: 0.773 (0.709–0.837) IA‐EI: 0.704 (0.631–0.777) IA‐N4PE: 0.679 (0.605–0.753)	*Shimadzu*: IP‐MS‐WashU: 0.872 (0.824–0.920) IP‐MS‐Shim: 0.825 (0.767–0.882) LC‐MS‐Arc: 0.775 (0.711–0.839) IA‐Elc: 0.773 (0.709–0.837) IA‐EI: 0.704 (0.631–0.777) IA‐N4PE: 0.679 (0.605–0.753)
							*University of Gothenburg*: WashU: 0.838 (0.785–0.891) IA‐Elc: 0.795 (0.738–0.853) LC‐MS‐Arc: 0.763 (0.700–0.827) IA‐N4PE: 0.706 (0.639–0.773) IA‐EI: 0.697 (0.628–0.767) IP‐MS‐UGOT: 0.678 (0.605–0.750) IA‐Quan: 0.636 (0.563–0.709)	*University of Gothenburg*: WashU: 0.814 (0.760–0.868) IA‐Elc: 0.728 (0.663–0.793) LC‐MS‐Arc: 0.742 (0.676–0.809) IA‐N4PE: 0.649 (0.577–0.721) IA‐EI: 0.667 (0.596–0.738) IP‐MS‐UGOT: 0.632 (0.557–0.707) IA‐Quan: 0.600 (0.525–0.675)
Zicha et al.^35^	*ADNI* AD, *n* = 18 MCI, *n* = 54	MCI AD	*n* = 49	Aβ‐_42/40_	IP‐MS Washington University ((IP‐MS‐WashU) Elecys Immunoassay Roche (IA‐Elc) IP‐MS–Shimadzu (IP‐MS‐Shim) IP‐MS–University of Gothenburg (IP‐MS‐UGOT) ADx Neurosciences Amyblood Simoa Quanterix Simoa	F^18^ florbetapir amyloid PET	PET IP‐MS WashU: 0.814 (0.736–0.892) IA‐EIc: 0.710 (0.617–0.803) IP‐MS‐Shim 0.715 (0.625–0.805) IP‐MS UGOT 0.643 (0.542–0.743) Amyblood Simoa 0.661 (0.563–0.760) Quanterix Simoa 0.645 (0.545–0.745)	
Meyer et al.^36^	PREVENT AD cohort At risk of AD *n* = 244 (PET cohort, *n* = 129)	At risk of AD	–	Aβ‐_42/40_	(1) Quanterix Simoa (2) IP‐MS	F^18‐^ NAV4694 Amyloid PET	PET IP‐MS: 0.808 (0.725–0.891) Simoa Quanterix: 0.756 (0.655–0.857)	
Keshavan et al.^37^	Dementia Free Population‐based Cohort (*n*=441, including 7 with MCI)	Not applicable	Not applicable	Aβ‐_42/40_	(1) Quanterix Simoa (2) LC‐MS	F^18^ Florbetapir Amyloid PET	PET LC‐MS: 0.817 (0.770–0.864) Simoa Quanterix: 0.620 (0.548–0.691)	
De Meyer et al.^38^	Longitudinal observational cohort. aMCI, *n* = 38	aMCI	*n* = 161	Aβ‐_42/40_	(1) ELISA (EUROIMMUN, Germany) (2) Simoa Amyblood platform (ADx NeuroSciences)	F^18^ Flutemetamol Amyloid PET in controls F^18^ Florbetaben Amyloid PET in aMCI	PET ELISA: 0.78 (0.72–0.84) Amyblood Simoa: 0.79 (0.73–0.85)	

Abbreviations: Aβ, amyloid beta; AD, Alzheimer's disease; ADNI, Alzheimer's Disease Neuroimaging Initiative; aMCI, amnestic mild cognitive impairment; AUC, area under the curve; CI, confidence interval; CSF, cerebrospinal fluid; ELISA, enzyme‐linked immunosorbent assay; IP, immunoprecipitation; LC, liquid chromatography; MCI, mild cognitive impairment; MS, mass spectrometry; PET, positron emission tomography; Simoa, single molecule protein detection assay.

**TABLE 2:** Summary of studies directly comparing plasma total tau or phosphorylated tau biomarker quantification techniques in AD.

Study	Patient cohort(s)	Disease setting	Cognitively normal controls	Tau biomarkers	Plasma biomarker methods compared	Reference standard	Reference standard AUC (95% CI)	
De Meyer et al.^38^	Longitudinal observational cohort. aMCI, *n* = 38	aMCI	*n* = 161	Aβ‐_42_/t‐tau ratio	(1) ELISA (EUROIMMUN, Germany) (2) Simoa Amyblood platform (ADx NeuroSciences)	F^18^ Flutemetamol Amyloid PET in controls F^18^ Florbetaben Amyloid PET in aMCI	Amyloid PET ELISA: 0.77 (0.71‐0.83) Amyblood Simoa: 0.77 (0.71–0.83)	
Bayoumy et al.^39^	AD, *n* = 40	AD	*n* = 40	p‐tau181 p‐tau217 p‐tau231	p‐tau181 (1) Simoa Eli Lilly (2) SImoa ADx Neurosciences (3) Simoa Quanterix p‐tau217 (1) Simoa Eli Lilly p‐tau231 (1) Simoa ADx Neurosciences (2) Simoa University of Gothenberg (UGOT)	Healthy age and sex matched controls	Healthy Controls vs AD p‐tau181 (1) Simoa Eli Lilly: 0.938 (0.872–1.000) (2) SImoa ADx: 0.988 (0.969–1.000) (3) Simoa Quanterix: 0.936 (0.885–0.987) p‐tau217 (1) Simoa Eli Lilly: 0.995 (0.987–1.000) p‐tau231 (1) Simoa ADx: 0.719 (0.607–0.831) (2) Simoa UGOT: 0.943 (0.896–0.991)	
Mielke et al.^40^	Mayo Clinic Study of Aging, Population‐based (*n*=200, including 23 with MCI)	Not applicable	Not applicable	p‐tau181 p‐tau217 p‐tau231	p‐tau181 (1) Simoa Quanterix (2) Eli Lilly MSD p‐tau217 Eli Lilly MSD p‐tau231 In‐house Simoa	Pittsburgh Compound B Amyloid PET F^18^ Flortaucipir Tau PET	Amyloid PiB‐PET p‐tau181 *Simoa Quanterix* All 0.77 (0.71–0.84) MCI 0.82 (0.63–1.00) *Eli MSD* All 0.79 (0.72–0.85) MCI 0.78 (0.58–0.98) p‐tau217 *Eli Lilly MSD* All 0.79 (0.72–0.85) MCI 0.82 (0.63–1.02) p‐tau231 *In‐house Simoa* All 0.73 (0.66–0.81) MCI 0.67 (0.39–0.95)	Tau‐PET p‐tau181 *Simoa Quanterix* All 0.73 (0.64–0.82) MCI 0.88 (0.66–1.09) *Eli MSD* All 0.81(0.73–0.89) MCI 0.86 (0.67–1.04) p‐tau217 *Eli Lilly MSD* All 0.82 (0.74–0.90) MCI 0.87 (0.67‐1.06) p‐tau231 *In‐house Simoa* All 0.78 (0.70–0.87) MCI 0.80 (0.54–1.06)
Groot et al.^41^	Cohort 1: *BioFinder Study* MCI, *n* = 25 Cohort 2: *Longitudinal cohort from Skane University, Sweden* MCI, *n* = 147	MCI	Cohort 1: *n* = 27	p‐tau217	p‐tau217 (1) Simoa Jansen (2) Simoa Lilly	Cohort 1: CSF Aβ_42_/Aβ_40_ F^18^ flutemetamol amyloid PET Cohort 2: CSF Aβ_42_/Aβ_40_	Cohort 1: CSF p‐tau217 Simoa Jansen 0.91 (0.84–0.99) Simoa Lilly 0.89 (0.80–0.98)	Cohort 1: Aβ PET p‐tau217 Simoa Jansen 0.91 (0.83–1.00) Simoa Lilly 0.90 (0.81–1.00)
							Cohort 2: CSF p‐tau217 Simoa Jansen 0.85 (0.79–0.91) Simoa Lilly 0.87 (0.82–0.93)	
Janelidze et al. Brain.^42^	MCI, *n* = 135 (*n* = 45 progressed to AD)	MCI AD	–	p‐tau181 p‐tau217 p‐tau231	p‐tau181 (1) Simoa ADx (2) IP‐MS WashU (3) MSD Lilly (4) Simoa UGOT (5) Lumipulse immuno assay by Fujirebo (6) Splex immunoassay from MSD p‐tau217 (1) IP‐MS WashU (2) MSD Lilly (3) Simoa Jansen p‐tau231 (1) Simoa UGOT	CSF Aβ_42_/Aβ_40_	CSF Aβ‐_40/42_ p‐tau181 (1) Simoa ADx 0.841 (0.768–0.913) (2) IP‐MS WashU 0.835 (0.765–0.906) (3) MSD Lilly 0.759 (0.676–0.841) (4) Simoa UGOT 0.743 (0.652–0.833) (5) Lumipulse immuno assay by Fujirebo 0.694 (0.604–0.784) (6) Splex immunoassay from MSD 0.642 (0.533–0.751) p‐tau217 (1) IP‐MS WashU 0.947 (0.907–0.987) (2) MSD Lilly 0.886 (0.827–0.944) (3) Simoa Jansen 0.858 (0.795–0.920) p‐tau231 (1) Simoa UGOT 0.784 (0.703–0.864)	Progression to AD from MCI p‐tau181 (1) Simoa ADx 0.846 (0.777–0.916) (2) IP‐MS WashU 0.835 (0.764–0.906) (3) MSD Lilly 0.813 (0.734–0.892) (4) Simoa UGOT 0.775 (0.692–0.858) (5) Lumipulse immuno assay by Fujirebo 0.735 (0.649–0.821) (6) Splex immunoassay from MSD 0.688 (0.579–0.796) p‐tau217 (1) IP‐MS WashU 0.932 (0.891–0.974) (2) MSD Lilly 0.889 (0.833–0.946) (3) Simoa Jansen 0.872 (0.814–0.931) p‐tau231 (1) Simoa UGOT 0.777 (0.699–0.856)

Abbreviations: Aβ, amyloid beta; AD, Alzheimer's disease; ADNI, Alzheimer's Disease Neuroimaging Initiative; aMCI, amnestic mild cognitive impairment; AUC, area under the curve; CI, confidence interval; CSF, cerebrospinal fluid; ELISA, enzyme‐linked immunosorbent assay; IP, immunoprecipitation; LC, liquid chromatography; MCI, mild cognitive impairment; MS, mass spectrometry; PET, positron emission tomography; p‐tau, phosphorylated tau; Simoa, single molecule protein detection assay.

### 3.1 Plasma Aβ_42/40_ in AD and MCI

**FIGURE 3:**
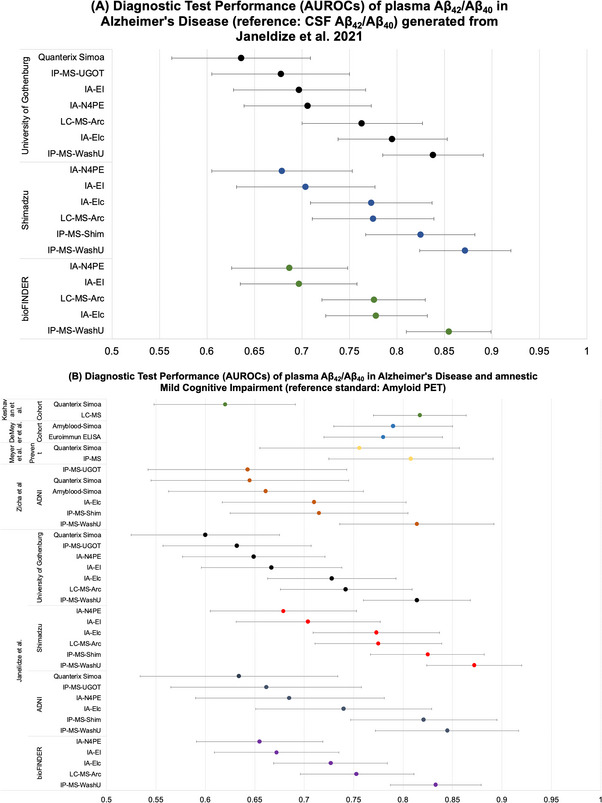
Diagnostic test performance of plasma Aβ_42_/Aβ_40_ in AD and MCI in reference to (A) CSF Aβ_42_/Aβ_40_ and (B) amyloid PET. IP‐MS Washington University (IP‐MS‐WashU); Elecys Immunoassay Roche (IA‐Elc); LC‐MS Araclon (LC‐MS‐Arc); Immunoassay Euroimmun (IA‐EI); Simoa immunoassay Quanterix (IA‐N4PE); IP‐MS–Shimadzu (IP‐MS‐Shim); IP‐MS–University of Gothenburg (IP‐MS‐UGOT); ADx Neurosciences Amyblood Simoa (Amyblood Simoa); Simoa immunoassay Quanterix (IA‐Quan). Aβ, amyloid beta; AD, Alzheimer's disease; ADNI, Alzheimer's Disease Neuroimaging Initiative; CSF, cerebrospinal fluid; IP, immunoprecipitation; LC, liquid chromatography; MCI, mild cognitive impairment; MS, mass spectrometry; PET, positron emission tomography; Simoa, single molecule protein detection assay.

The test performance of various methods to quantify Aβ_42/40_ in five comparative studies are displayed in Table 1. Figure 3 depicts the main AUC point estimates and corresponding 95% CIs extracted from these studies for the performance of various plasma Aβ_42/40_ techniques in relation to (1) CSF Aβ_42/40_ and (2) amyloid‐PET reference standards.

Janelidze et al.^34^ examined 286 individuals from the Swedish BioFINDER study with head‐to‐head comparisons of five different plasma Aβ_42/40_ assays. An additional three plasma Aβ_42/40_ assays were examined in a validation cohort and two subcohorts. This study found that the IP‐MS technique from Washington University demonstrated significantly higher accuracy for discriminating the presence of AD on the basis of CSF Aβ_42/40_ (AUC = 0.855 [95% CI: 0.810–0.899]) and F^18^ flutemetamol PET (AUC = 0.833 [95% CI: 0.787–0.879]) compared to liquid chromatography mass spectrometry, Elecys immunoassays, immunoassays from Euroimmun, and Simoa N4PE immunoassays from Quanterix.^34^ Janelidze et al.^34^ replicated findings in three additional cohorts: an Alzheimer Disease Neuroimaging Initiative (ADNI) cohort (*n* = 122) as well as two subcohorts from Shimadzu, Kyoto, Japan (*n* = 200) and a cohort from the University of Gothenburg, Gothenburg, Sweden (*n* = 227).^34^ The test with the poorest performance was the N4PE Simoa Quanterix Aβ_42/40_ assay, which could discriminate AD based on CSF Aβ_42/40_ and Aβ PET with AUCs of 0.687 (95% CI: 0.626–0.748) and 0.655 (95% CI: 0.591–0.719), respectively.^34^ An additional Simoa Quanterix (IA‐Quan) assay and IP‐MS method from the University of Gothenburg were evaluated in a subcohort of 227 and the AUCs reported for CSF Aβ_42/40_ and Aβ PET for both techniques were low (see Table 1 for details).

Another study group, Zicha et al.,^35^ agnostic to the technology used to quantify Aβ, systematically selected the top 6 of 15 methods to quantify plasma Aβ_42_ and Aβ_40_. After the selection process Zicha et al.^35^ compared six assays (three ligand binding methods and three mass spectrometry–based methods) head to head on their ability to predict amyloid PET positivity.^35^ In this sample of 121 ADNI participants, the IP‐MS assay from Washington University Aβ_42/40_ had an AUC of 0.814 (95% CI: 0.736–0.892), the Elecsys Cobas e601 immunoassay by Roche had an AUC of 0.710 (95% CI: 0.617–0.803), the IP‐MS technique from Shimadzu had an AUC of 0.715 (95% CI: 0.625–0.805), an IP‐MS technique from University of Gothenburg had an AUC of 0.643 (95% CI: 0.542–0.743), the ADx Neurosciences Amyblood SIMOA technique had an AUC of 0.661 (95% CI: 0.563–0.760), and Simoa Quanterix had an AUC of 0.645 (95% CI: 0.545–0.745).^35^ A third comparison study by Meyer et al.^36^ compared plasma Aβ_42/40_ quantified through Simoa Quanterix and IP‐MS and noted similar accuracy in predicting Aβ PET positivity. While Simoa Quanterix had an AUC of 0.756 (95% CI: 0.655–0.857), IP‐MS had an AUC of 0.808 (95% CI: 0.725–0.891).^36^ Another study, by Keshavan et al.,^37^ in MCI and dementia‐free participants compared the discriminative performance of an Aβ_42/40_ Simoa Quanterix assay to a liquid chromatography‐tandem mass spectrometry (LC‐MS) assay to discern amyloid‐positive PET. While the Simoa Aβ_42/40_ method had an AUC of 0.620 (95% CI: 0.548–0.691), the LC‐MS Aβ_42/40_ had an AUC of 0.817 (95% CI: 0.770–0.864).^37^ The final comparison study by De Meyer et al.^38^ compared an ELISA technique to a Simoa Amyblood platform and found that the receiver operator characteristic AUC did not vary between ELISA 0.78 (95% CI: 0.72–0.84) and Simoa Amyblood 0.79 (95% CI: 0.73 to 0.85). Furthermore, both methods had negative predictive values of >88%.^38^

The Simoa Amyblood method, mentioned above, uses a more specific antibody, which quantifies the full length of Aβ_42_ and Aβ_40_ and not fragments, and which ultimately improves the specificity of this assay.^38^, ^43^ While one study found that the Simoa Amyblood method could discern AD on Amyloid PET with an AUC of 0.79 (95% CI: 0.73–0.85),^38^ Zicha et al., in an ADNI cohort, found an AUC of 0.66 (95% CI: 0.56–0.76) for this method.^35^ Another recent study used a modified Simoa technique to quantify plasma Aβ_42/40_ from plasma neuronal derived extracellular vesicles and noted an AUC of 0.96 (95% CI: 0.92–0.99) discerning AD based on amyloid PET.^44^

### 3.2 Plasma tau in AD and MCI

**FIGURE 4:**
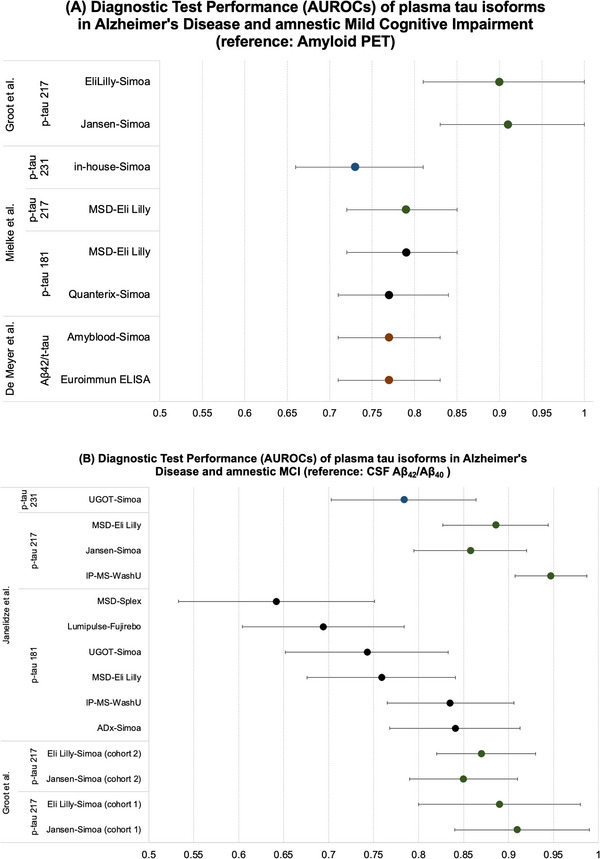
Diagnostic test performance of plasma tau isoforms in AD and MCI in reference to (A) CSF Aβ_42_/Aβ_40_ and (B) amyloid PET. University of Gothenburg Simoa (UGOT‐Simoa); Meso Scale Discovery platform from Eli Lilly (MSD‐Eli Lilly); IP‐MS Washington University ((IP‐MS‐WashU); Splex immunoassay from Meso Scale Discovery platform (MSD‐Splex); Lumipulse immuno assay by Fujirebo (Lumipulse‐Fujirebo); ADx Neurosciences Simoa (ADx Simoa); Simoa from Eli Lilly (EliLilly‐Simoa); Simoa from Jansen (Jansen‐Simoa); Simoa from Quanterix (Qunaterix Simoa)ADx Neurosciences Amyblood Simoa (Amyblood Simoa); and ELISA from EUROIMMUN, Germany (Euroimmun ELISA). Aβ, amyloid beta; AD, Alzheimer's disease; ADNI, Alzheimer's Disease Neuroimaging Initiative; CSF, cerebrospinal fluid; IP, immunoprecipitation; LC, liquid chromatography; MCI, mild cognitive impairment; MS, mass spectrometry; PET, positron emission tomography; Simoa, single molecule protein detection assay.

The main AUC point estimates and corresponding 95% CIs extracted from studies evaluating the performance of various plasma tau techniques across tau isoforms in relation to CSF Aβ_42/40_ and amyloid PET reference standards are depicted in Table 2 and Figure 4.

With respect to tau‐based assays, De Meyer et al.^38^ did not directly compare t‐tau individually, but rather compared an ELISA to Simoa Aβ_42_/t‐tau ratio. In CSF it has been reported that Aβ_42_/t‐tau is a better predictor of progression in AD than the Aβ_42/40_ ratio alone.^38^ As displayed in Table 2, the AUC for both methods for the Aβ_42_/t‐tau ratio was identical. In another study, Bayoumy et al.^39^ compared six different Simoa assays for three different p‐tau biomarkers (p‐tau181, p‐tau217, p‐tau231) and found overall excellent test performance for nearly all Simoa techniques with nearly all AUCs > 0.9. However, one important limitation of this study is that their diagnostic reference standard was age‐ and sex‐matched controls. This also makes the AUCs reported here very difficult to compare to other studies that have evaluated test performance in relation to a diagnostic reference standard such as PET or CSF analysis.

Another study, by Mielke et al.,^40^ evaluated a cohort of 200 participants and compared two p‐tau Simoa methods and a Meso Scale Discovery (MSD) Platform (Eli Lilly) against both Pittsburgh compound B (PiB) amyloid PET and F^18^ flortaucipir tau PET. The MSD works like an ELISA and uses electrochemiluminescence.^40^ Mielke et al.^40^ evaluated p‐tau231 and p‐tau217, but they did not compare two different quantification methods head to head for these two biomarkers. However, Mielke et al.^40^ did compare two different p‐tau181 methods and found that with respect to amyloid PET in patients with MCI, Simoa Quanterix had an AUC of 0.82 (95% CI: 0.63–1.00), while Eli Lilly MSD had an AUC of 0.78 (95% CI: 0.58–0.98). With respect to tau PET, in patients with MCI Simoa Quanterix had an AUC of 0.88 (95% CI: 0.66–1.09) while Eli Lilly MSD had an AUC of 0.86 (95% CI: 0.67–1.04). The very small sample size of MCI patients in this study likely contributed to the wide confidence limits. Differences between Simoa Quanterix and Eli Lilly MSD may have arisen due to differences in the antibodies used in each platform.^40^

Groot et al.^41^ directly compared two p‐tau217 Simoa assays from Eli Lilly and Janssen Research and Development and demonstrated excellent AUCs in two independent cohorts (see Table 2). Finally, perhaps the highest quality evidence found for head‐to‐head comparisons of various plasma p‐tau techniques was by Janelidze et al.^42^ examining p‐tau181, p‐tau217, and p‐tau231 across 10 plasma assays including IP‐MS‐ and Simoa‐based methods in 135 patients with MCI followed for 4.9 years.^42^ The results from Janelidze et al.^42^ are summarized in Table 2 and Figure 4 and demonstrate good to excellent AUCs for most IP‐MS‐ and Simoa‐based methods at detecting AD based on CSF Aβ_40/42_ and in discriminating those who progress from MCI to AD.

### 3.3 Plasma NfL and GFAP in AD

This narrative review did not identify any comparative analyses of different plasma methods to quantify NfL or GFAP biomarkers. While both are elevated in CSF and plasma in AD, there has been variable diagnostic test performance of NfL and GFAP reported in the literature to date with most studies measuring the performance of these biomarkers in reference to a clinical diagnosis of AD and fewer evaluating these in reference to CSF amyloid or PET markers.

Studies of NfL suggest that it may have some value in discriminating AD from controls without neurodegenerative diseases. In one study using data from ADNI, a Quanterix Simoa assay was used to evaluate plasma NfL and discriminated the AD dementia group from normal controls with an AUC = 0.87, while CSF Aβ_42_ had an AUC = 0.88 and CSF t‐tau had an AUC = 0.90 (95% CIs were not reported).^45^ Another study evaluated several plasma biomarkers, including NfL measured with a Quanterix Simoa assay in reference to a pathological diagnosis of AD and noted that plasma Aβ_42/40_ (AUC = 0.58, 95% CI: 0.47–0.68) and NfL (AUC = 0.59, 95% CI: 0.48–0.70) had the lowest diagnostic test performance, while p‐tau181 (AUC = 0.77, 95% CI: 0.67–0.87) and p‐tau217 (AUC = 0.84, 95% CI: 0.75–0.92) had the best diagnostic test performance.^46^ Two studies, both using a Quanterix Simoa assay, compared NfL to amyloid PET signal but produced conflicting results with one study finding that NfL predicted amyloid PET positivity (AUC = 0.76, 95% CI not reported) while the other study found that it did not (AUC = 0.59, 95% CI: 0.47–0.71).^36^, ^47^

A few studies to date have evaluated the diagnostic test performance of GFAP in AD. In one study, plasma GFAP concentration was elevated in PET amyloid positive patients compared to those who were PET amyloid negative,^15^ AUC = 0.761, which was superior to CSF GFAP AUC = 0.694 (95% CIs not reported).^15^ This is consistent with another study in which the diagnostic test performance of plasma GFAP was greater than CSF GFAP and the change in the magnitude of plasma GFAP was greater than changes in CSF GFAP in AD.^48^ This study also demonstrated elevations in plasma GFAP in pre‐clinical AD and in those with symptomatic AD. Similarly, CSF amyloid positive status is associated with plasma GFAP (AUC = 0.72, 95% CI: 0.70–0.74) and NfL (AUC = 0.60, 95% CI: 0.57–0.64), although not as strongly as plasma Aβ_42/40_ (AUC = 0.79, 95% CI: 0.76–0.82) and plasma p‐tau 217 (AUC = 0.82, 95% CI: 0.80–0.85).^49^ Plasma GFAP has been reported to predict incident clinical diagnoses of AD at 0 to 17 years with an AUC = 0.729 (95% CI: 0.682–0.776), which was better than plasma p‐tau181 (AUC = 0.610, 95% CI: 0.556–0.776) and plasma NfL (AUC = 0.676, 95% CI: 0.629–0.724).^50^

There are some studies comparing NfL between AD and other neurodegenerative or neurological conditions that can cause later life cognitive decline, an important comparison as these are conditions that would have to be differentiated from AD in clinical practice. Plasma NfL is elevated across multiple neurodegenerative conditions including AD, amnestic MCI, and dementia with Lewy bodies (DLB), but is especially elevated in frontotemporal dementia and amyotrophic lateral sclerosis.^51^ Plasma GFAP has mostly been evaluated in AD, though one study also reported elevations in plasma GFAP in DLB^52^ and a recent study reported elevations in plasma GFAP in the cerebellar subtype of multi‐system atrophy.^53^ More studies are needed to directly compare plasma GFAP across neurodegenerative disorders. As markers of neuronal and glial injury that are elevated in other central nervous system diseases, NfL and potentially GFAP may prove to be better predictors of disease stage or prognosis than the type of disease although the evidence is more robust for NfL than it is for GFAP at present.^54^, ^55^, ^56^, ^57^, ^58^, ^59^, ^60^ However, it is worth exploring whether levels of NfL or GFAP, in combination with more specific markers of disease processes, can improve diagnostic accuracy.

## 4 STUDIES OF CORE CSF AND PLASMA BIOMARKERS IN CAA

Our MEDLINE/Pubmed search yielded 425 titles and abstracts. The articles extracted and reviewed in full are detailed below for CSF and plasma biomarkers of CAA.

### 4.1 CSF

Overall, two meta‐analyses and six case control studies were identified, evaluating the core CSF biomarkers of Aβ_42_, Aβ_40_, p‐tau, and t‐tau in relation to Boston criteria probable or possible CAA. After the time frame of our literature search, we found one additional study recently published, comparing cohorts of CAA, AD, MCI related to AD, and normal controls. These nine studies are summarized in Table 3.^61^, ^62^, ^63^, ^64^, ^65^, ^66^, ^67^, ^68^ Some of these studies are limited by small sample sizes.

**TABLE 3:** Summary of studies in CAA evaluating the core CSF biomarkers (Aβ_42_, Aβ_40_, p‐tau, and t‐tau in relation to Boston criteria probable or possible CAA).

Study	Design	Study participants	CSF biomarkers evaluated	Methods of biomarker quantification	Main findings
Sembill et al.^61^	Case control	CAA, *n* = 67 AD, *n* = 76 MCI due to AD, *n* = 75 MCI unlikely from AD, *n* = 76 Healthy controls, *n* = 78	Aβ_42_ Aβ_40_ Aβ_42_/Aβ_40_ p‐tau t‐tau	ELISA	CAA and healthy controls: ⚬ Aβ_42_ AUC = 0.95 (95% CI: 0.92–0.99) ⚬ Aβ_40_ AUC = 0.96 (95%CI: 0.93–1.00) CAA and AD: ⚬ Aβ_42_ AUC = 0.75 (95% CI: 0.67–0.83) ⚬ Aβ_40_ AUC = 0.76 (95% CI: 0.68–0.84) CAA and all controls (AD, MCI, healthy controls): ⚬ Aβ_42_ AUC = 0.82 (95% CI: 0.75–0.88) ⚬ Aβ_40_ AUC = 0.83 (95% CI: 0.76–0.89)
Verbeek et al.^62^	Case control	Probable CAA, *n* = 17 AD, *n* = 72 Controls, *n* = 58	Aβ_42_ Aβ_40_ Aβ_42_/Aβ_40_ p‐tau t‐tau	ELISA	CAA and controls: (95% CI not reported): ⚬ Aβ_42_ AUC = 0.96 ⚬ Aβ_40_ AUC = 0.76 ⚬ tau AUC = 0.73 ⚬ p‐tau181 AUC = 0.67 CAA and AD: (95% CI not reported): ⚬ Aβ_42_ AUC = 0.68 ⚬ Aβ_40_ AUC = 0.74 ⚬ tau AUC = 0.80 ⚬ p‐tau181 AUC = 0.79
Grangeon et al.^63^	Case control	Probable CAA, *n* = 63 AD, *n* = 27 Controls, *n* = 21	Aβ_42_ Aβ_40_ Aβ_42_/Aβ_40_ p‐tau t‐tau	ELISA	CAA and controls: ⚬ Aβ_42_/Aβ_40_ AUC = 0.62 (95% CI: 0.43–0.81) ⚬ Aβ_42_ AUC = 0.79 (95% CI: 0.66–0.92) ⚬ Aβ_40_ AUC = 0.69 (95% CI: 0.51–0.87) ⚬ tau AUC = 0.60 (95% CI: 0.43–0.77) ⚬ p‐tau AUC = 0.60 (95% CI: 0.44–0.76) CAA and AD: ⚬ Aβ_42_/Aβ_40_ AUC = 0.74 (95% CI: 0.62–0.86) ⚬ Aβ_42_ AUC = 0.62 (95% CI: 0.49–0.76) ⚬ Aβ_40_ AUC = 0.72 (95% CI: 0.60–0.85) ⚬ Tau AUC = 0.68 (95% CI: 0.53–0.82) ⚬ p‐tau AUC = 0.69 (95% CI: 0.56–0.83)
Banerjee et al.^64^	Case control	Probable CAA, *n* = 10 AD, *n* = 20 Controls, *n* = 10	Aβ_42_ Aβ_40_ Aβ_42_/Aβ_40_ p‐tau t‐tau	Electrochemiluminescence Aβ ELISA for tau	No ROC analyses reported AD patients had higher median t‐tau and p‐tau than in control or CAA cohorts Patients with CAA had lower median Aβ_42_, Aβ_40_, Aβ_38_ even after adjusting for age
Renard et al.^65^	Case control	Probable or possible CAA, *n* = 13 AD, *n* = 42 Controls, *n* = 14	Aβ_42_ Aβ_40_ Aβ_42_/Aβ_40_ p‐tau t‐tau	ELISA	CAA and controls (95% CI not reported): ⚬ Aβ_42_, AUC = 0.75 ⚬ Aβ_40_, AUC = 0.64 ⚬ t‐tau, AUC = 0.78 ⚬ ptau, AUC = 0.62 CAA and AD (95% CI not reported): ⚬ Aβ_42_, AUC = 0.56 ⚬ Aβ_40_, AUC = 0.80 ⚬ t‐tau, AUC = 0.77 ⚬ ptau, AUC = 0.93
Martinez‐Lizana et al.^66^	Case control	CAA ⚬ without cSAH, *n* = 12 ⚬ with cSAH +/– ICH, *n* = 7 AD, *n* = 44 Controls, *n* = 20	Aβ_42_ Aβ_40_ Aβ_42_/Aβ_40_ p‐tau t‐tau	ELISA	No ROC analyses reported Patients with CAA (with and without cSAH) had reductions in CSF Aβ_42_ and Aβ_40_ compared to AD and normal controls T‐tau and p‐tau levels were significantly lower in CAA than in those with AD
Margraf et al.^67^	Case control	Probable CAA, *n* = 31 AD, *n* = 28 Controls, *n* = 30	Aβ_42_ Aβ_40_ Aβ_42_/Aβ_40_ p‐tau t‐tau	Electrochemiluminescence	CAA and controls: ⚬ Aβ_42_/Aβ_40_ AUC = 0.88 (95% CI: 0.79–0.97) ⚬ Aβ_42_ AUC = 0.86 (95% CI: 0.76–0.96) ⚬ Aβ_40_ AUC = 0.63 (95% CI: 0.49–0.77) ⚬ tau AUC = 0.85 (95% CI: 0.75–0.94) ⚬ p‐tau181 AUC = 0.82 (95% CI: 0.71–0.93) CAA and AD: ⚬ Aβ_42_/Aβ_40_ AUC 0.61 (95% CI: 0.46–0.75) ⚬ Aβ_42_ AUC = 0.54 (95% CI: 0.39–0.7) ⚬ Aβ_40_ AUC = 0.58 (95% CI: 0.43–0.73) ⚬ tau AUC = 0.68 (95% CI: 0.54–0.82) ⚬ p‐tau181, AUC = 0.75 (95% CI: 0.61–0.88)
Margraf et al.^67^	Meta‐Analysis	Pooled estimates of: Verbeek et al.^62^; Martinez‐Linzana et al. 2015; Renard et al.^65^; Banerjee et al.^64^; and Margraf et al.^67^	Aβ_42_ Aβ_40_ Aβ_42_/Aβ_40_ p‐tau t‐tau	Pooled across methods above	CAA and controls: ⚬ Aβ_42_/Aβ_40,_ AUC = 0.90 (95% CI: 0.86–0.94) ⚬ Aβ_42_, AUC = 0.89 (95% CI: 0.84–0.94) ⚬ Aβ_40_, AUC = 0.76 (95% CI: 0.69–0.82) ⚬ t‐tau, AUC = 0.79 (95% CI: 0.73–0.85) ⚬ ptau181, AUC = 0.71 (95% CI: 0.63–0.78) CAA and AD: ⚬ Aβ_42_/Aβ_40_, AUC = 0.69, (95% CI: 0.63–0.76) ⚬ Aβ_40_, AUC = 0.73 (95% CI: 0.67–0.79) ⚬ Aβ_42_, AUC = 0.54 (95% CI: 0.47‐0.61) ⚬ ptau181, AUC = 0.76 (95% CI: 0.69–0.82) ⚬ t‐tau, AUC = 0.71 (95% CI: 0.64–0.77)
Charidimou et al.^68^	Meta‐Analysis	Pooled estimates of: Verbeek et al. ^62^; Martinez‐ Lizana et al.^66^; Renard et al.^65^	Aβ_42_ Aβ_40_ Aβ_42_/Aβ_40_ p‐tau t‐tau	Pooled across methods above	CAA and controls ratio of means: ⚬ Aβ_42_ 0.49 (95% CI: 0.38–0.64), *P < 0.0001* ⚬ Aβ_40_ 0.70 (95% CI: 0.63–0.78), *P < 0.0001* ⚬ t‐tau 1.54 (95% CI: 1.15–2.07) *P* = 0.004 ⚬ p‐tau 1.24 (95% CI: 0.99–1.54), *P* = 0.06 CAA and AD ratio of means: ⚬ Aβ_42_ 1.0 (95% CI: 0.81–1.23), *P =* 0.97 ⚬ Aβ_40_ 0.76 (95% CI: 0.69–0.83), *P* < 0.0001 ⚬ t‐tau 0.63 (95% CI: 0.54–0.74), *P* < 0.0001 ⚬ p‐tau 0.60 (95% CI: 0.50–0.71), *P* < 0.0001

Abbreviations: Aβ, amyloid beta; AD, Alzheimer's disease; ADNI, Alzheimer's Disease Neuroimaging Initiative; aMCI, amnestic mild cognitive impairment; AUC, area under the curve; CAA, cerebral amyloid angiopathy; CI, confidence interval; cSAH, convexity subarachnoid hemorrhage; CSF, cerebrospinal fluid; ELISA, enzyme‐linked immunosorbent assay; ICH, intracerebral hemorrhage; IP, immunoprecipitation; LC, liquid chromatography; MCI, mild cognitive impairment; MS, mass spectrometry; MSD, Meso Scale Discovery platform; PET, positron emission tomography; p‐tau, phosphorylated tau; Simoa, single molecule protein detection assay; t‐tau, total tau.

A meta‐analysis by Charidimou et al.^68^ pooled results from three studies,^62^, ^65^, ^66^ with a total of 59 patients with CAA, 158 patients with AD, and 94 HC. These studies are also independently summarized in Table 3.^62^, ^65^, ^66^ In this meta‐analysis three core CSF biomarkers differentiated CAA from normal controls: CSF Aβ_42_ (ratio of means [RoM] = 0.49 [95% CI: 0.38–0.64]), CSF Aβ_40_ (RoM = 0.70 [95% CI: 0.63–0.78]), and t‐tau (RoM = 1.54 [95% CI: 1.15–2.07]), but not p‐tau.^68^ With respect to the discrimination between CAA and AD, CSF Aβ_40_ RoM = 0.76 (95% CI: 0.69–0.83) was the most discriminative, while no difference in CSF Aβ_42_ was noted.^68^

Since this meta‐analysis by Charidimou et al.^68^ was published in 2018, three additional case–control studies have been published,^63^, ^64^, ^67^ one of which also includes an updated meta‐analysis.^67^ A larger case–control study of modified Boston criteria probable CAA patients is reported in Grangeon et al.^63^ In this study authors used ELISA to evaluate CSF Aβ_42_, Aβ_40_, p‐tau, and t‐tau between 63 patients with CAA, 27 patients with AD, and 21 controls.^63^ Overall, 85% of patients who had probable CAA according to revised Boston criteria had abnormal CSF biomarkers.^63^ Patients with CAA and AD had similar levels of Aβ_42_, but overall, CAA had lower levels of CSF t‐tau, p‐tau, and Aβ_40_. That said, three CSF profiles were identified among those with CAA: (1) an AD‐like profile with elevated tau and p‐tau levels (50.8%), (2) an isolated reduction in Aβ_42_ (34.9%), and (3) normal Aβ_42_ (14.3%).^63^ Across all three CAA profiles, however, a reduction in Aβ_40_ was uniformly a distinguishing characteristic compared to AD cases.^63^ Margraf et al.,^67^ report a case–control study of 31 patients with CAA, 28 patients with AD, and 30 normal controls, found that Aβ_42/40_ provided the best discrimination between CAA and controls AUC = 0.88 (95% CI: 0.79–0.97), followed by tau AUC = 0.85 (95% CI: 0.75–0.94) and p‐tau181 AUC = 0.82 (95% CI: 0.71–0.93). For the discrimination between CAA and AD, p‐tau181 had the best test performance, AUC = 0.75 (95% CI: 0.61–0.88), while other biomarkers did not.^67^

**FIGURE 5:**
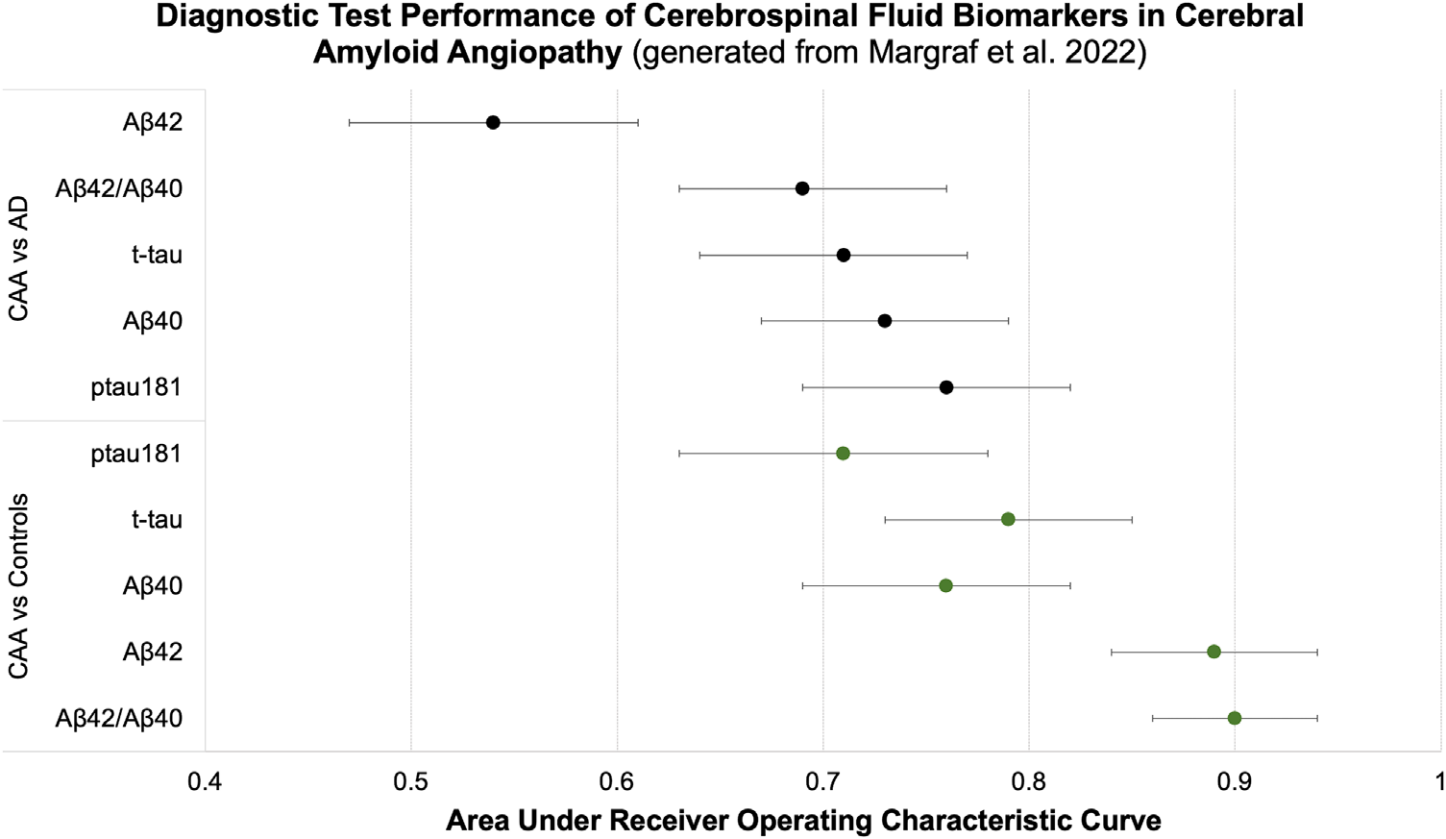
Diagnostic test performance of cerebrospinal fluid biomarkers in cerebral amyloid angiopathy. Figure generated from data extracted from Margraf et al.^67^

Margraf et al.^67^ included their own case–control data and conducted a meta‐analysis of four additional studies.^62^, ^64^, ^65^, ^66^ In this most recent meta‐analysis, summarized in Figure 5, Aβ_42/40_ best discriminated between CAA and controls, AUC = 0.90 (95% CI: 0.86–0.94), while p‐tau181 had the best discriminative performance between CAA and AD, AUC = 0.76 (95% CI: 0.69–0.82). Overall, in this analysis the core biomarkers did not distinguish as well between CAA and AD cases as they did between CAA and normal controls.

The most recently published, and now the largest case control study, evaluated CSF biomarkers (Aβ_42_, Aβ_40_, p‐tau, and t‐tau) in those with possible or probable CAA (*n* = 67) and compared them to HC (*n* = 78), AD (*n* = 76), MCI likely from AD (*n* = 75), and MCI unlikely from AD (*n* = 76).^61^ The difference of means (DoM) in the concentration of CSF Aβ_40_ in reference to HC was greatest for those with CAA (−22.5% [95% CI: −28.9% to −16.1%]) compared to other groups including AD (−9.3% [95% CI: −15.7 to −2.9]), MCI due to AD (+4.9% [95% CI: −2.3% to 12.1%]), and MCI not due to AD (−7.6% [95% CI: −14.5% to −0.7%]). Overall there were comparable CSF Aβ_42_ concentrations among CAA, AD, and MCI due to AD, though Aβ_42_ was reduced compared to HC.^61^ While CSF p‐tau and t‐tau were elevated in CAA compared to HC and MCI not due to AD, they were reduced relative to those with AD and those with MCI related to AD. Overall, these results are congruent with prior work suggesting that evaluating Aβ_42_, Aβ_40_, p‐tau, and t‐tau in combination may help discern among CAA, AD, MCI not due to AD, and HC.^61^ As displayed in Table 3, CSF Aβ_40_ and Aβ_42_ provide excellent discriminative ability for the diagnosis of CAA compared to HC and good discriminative ability for the diagnosis of CAA compared to all controls.

### 4.2 Plasma

Few studies have evaluated plasma biomarkers in CAA and there is far greater heterogeneity among the studies that have. It is difficult to predict, therefore, what the plasma profile of CAA may look like and whether this will coincide with previously reported CSF profiles of CAA. While the plasma biomarker profiles of AD appear to coincide with AD CSF biomarker profiles, the same cannot be assumed for CAA. One hypothesis is that because Aβ_40_ is sequestered in cerebral vasculature it might be concordantly reduced in both plasma and CSF. However, one could also surmise that Aβ_40_ might be increased in plasma, because with the accumulation of Aβ_40_ within cerebral small vessel walls it may be sequestered from CSF but could spill over into the systemic circulation, particularly given that CAA is associated with vessel wall disruption and hemorrhage. Furthermore, Aβ_40_ may decrease in the early pre‐symptomatic phase and then increase in the symptomatic hemorrhage phase. Studies to further evaluate these hypotheses are needed.

In one of the first studies using ELISA to quantify plasma Aβ_40_ and Aβ_42_, between 25 patients with probable or definitive CAA related intracerebral hemorrhages (ICH) and 21 patients with hypertensive ICH, no differences in plasma Aβ_40_ and Aβ_42_ were noted.^69^ In Piccarducci et al.^70^ in a cohort of 20 CAA patients and 20 HC, an in‐house immunoenzymatic assay was used to quantify the plasma Aβ_42/40_ ratio. The plasma Aβ_42/40_ ratio was decreased in CAA compared to HC, driven by a higher plasma Aβ_40_, in CAA compared to HC.^70^ The mean Aβ_40_ detected in serum of CAA patients was 5.92 ± 1.41 pg/mg and in normal controls it was 4.13 ± 1.28pg/mg, a difference significant at *P* = 0.0002.^70^ Another study using multiplexing technology with detecting microspheres found that plasma Aβ_42_, truncated fragments of Aβ_40_, and full‐length Aβ_40_ were elevated in patients with CAA‐related intracerebral hemorrhage compared to HC.^71^

Using a highly sensitive dielectrophoretic driven biosensor, Kim et al.^72^ quantified Aβ_40_ and Aβ_42_ in 25 persons without AD and 19 with AD. These participants were further classified as probable CAA according to modified Boston criteria. Among those with probable CAA, Aβ_40_ was elevated compared to those without CAA and Aβ_40_ discriminated probable CAA from AD with an AUC = 1.0 (95% CI: 1.0–1.0).^72^ The findings from these studies support the hypothesis that amyloid deposition in the vasculature causes spillover into the systematic circulation.

In contrast, a study using Simoa to measure plasma Aβ in pre‐symptomatic Dutch type hereditary CAA found that both Aβ_40_ and Aβ_42_ were reduced in mutation carriers (*n* = 9) compared to non‐carriers (NC; *n* = 8).^73^ This was the only identified study in which CAA cases were confirmed by genetic or neuropathological data, and thus the diagnosis of CAA in this study was probably more accurate. These data also raise the possibility that plasma Aβ_40_ concentrations could vary by CAA disease stage, with low Aβ_40_ in the pre‐symptomatic CAA phase followed by high Aβ_40_ in the symptomatic phase when there is vessel wall breakdown and hemorrhage. A recent disease stage framework for CAA has been published and it would be interesting for future studies to evaluate whether there might be variations in plasma Aβ concentrations according to stage.^74^

Finally, another study examined the association of plasma Aβ_40_ concentrations, measured by ELISA, with white matter hyperintensities in patients with AD, MCI, and CAA.^75^ In this study, plasma Aβ_42_ concentrations were lower in AD and MCI cohorts than in CAA, but Aβ_40_ concentration was similar across the three groups.^75^ However, there was no control group so the study could not conclude whether Aβ_40_ and Aβ_42_ differed between CAA and persons without MCI or AD dementia.

### 4.3 CAA‐related inflammation

CAA‐related inflammation (CAA‐ri) is an autoimmune disorder characterized by the production of antibodies against Aβ deposited within the walls of cortical and leptomeningeal vessels.^76^, ^77^ This autoimmune response against vascular amyloid induces a neuroinflammatory response that can result in new headache, neuropsychiatric manifestations, focal neurologic deficit, hemorrhage, subacute leukoencephalopathy, seizure, coma, and/or death.^76^, ^77^, ^78^ While CAA‐ri is associated with similar neuroimaging characteristics depicted in Figure 1 such as microbleeds, macro‐hemorrhages, and superficial siderosis, some of the differentiating neuroimaging hallmarks that can occur with CAA‐ri include: (1) acute and confluent T2 fluid attenuated inversion recovery hyperintensities affecting subcortical U‐fibers manifesting as a result of acute cerebral vasogenic edema, (2) mass effect, and (3) contrast enhancement of the leptomeninges or parenchyma.^78^, ^79^, ^80^ CAA‐ri is characterized by perivascular inflammation with or without frank vasculitis. When vasculitis is present it is has also been termed Aβ related angiitis (ABRA).^81^ The neuroimaging features of hemorrhage and edema in CAA‐ri are similar to the amyloid‐related imaging abnormalities (ARIA) seen in those receiving amyloid immunotherapies in AD: ARIA‐edema (ARIA‐E) and ARIA‐hemorrhage (ARIA‐H).^82^, ^83^, ^84^, ^85^ CAA‐ri, therefore, has been proposed as a naturally occurring model of amyloid immunotherapy–induced ARIA.^77^, ^86^

It is important for clinicians to recognize CAA‐ri as many patients appear to respond to immunosuppressive agents including glucocorticoids.^77^, ^86^, ^87^ Advances in understanding the evidence‐based management of CAA‐ri may help inform the appropriate approach to managing ARIA‐E and ARIA‐H as well, which will become more common as lecanemab and donanemab enter clinical practice. In the largest registry of 113 participants with CAA‐ri treated with immunosuppression, 70.3% of participants had clinical recovery and 45.1% had radiographic recovery at 3 months, with an increase in radiologic recovery to 77.4% at 1 year.^77^ CAA‐ri recurred in 38.3% in the subsequent 24 months, and recurrence was more likely if oral corticosteroid tapering occurred rapidly.^77^

Definitive diagnosis of CAA‐ri requires biopsy with neuropathological confirmation. However, it is increasingly common for patients to be diagnosed without biopsy, using validated neuroradiological criteria.^76^ Emerging evidence suggests that fluid markers may prove to be useful in the diagnosis and monitoring of CAA‐ri. Using an ultrasensitive ELISA assay with magnetic beads, elevated anti‐amyloid autoantibody levels have been detected in CSF during the active phases of CAA‐ri, which decline to near normal levels during the remission phases of CAA‐ri in response to immunosuppression.^86^ Furthermore, the concentration of CSF anti‐amyloid autoantibodies was positively correlated with the concentration of CSF Aβ_40_ and Aβ_42_ in this study, although this correlation was stronger with the soluble isoform Aβ_40_.^86^ Small case series using amyloid PET suggests that, in CAA‐ri, there is reduced amyloid deposition in regions with the greatest CAA‐ri associated cerebral edema—perhaps suggesting that the neuroinflammatory response facilitates the removal of cerebral amyloid.^88^ In another study, elevations in CSF anti‐amyloid autoantibodies were noted in those with CAA‐ri but not in those patients with either AD or MCI who had multiple cerebral microbleeds or confluent white matter hyperintensities, suggesting that the presence of anti‐amyloid autoantibodies in CSF may be a specific marker of CAA‐ri and not merely a consequence of asymptomatic concomitant CAA.^89^

While CSF anti‐amyloid antibodies may be a biomarker of CAA‐ri, there are no reported studies, to the best of our knowledge, evaluating whether specific plasma anti‐amyloid antibodies may distinguish CAA‐ri from CAA without inflammation and/or AD. One hypothesis, supported by one cohort study, is that CSF anti‐amyloid antibodies in CAA‐ri are intrathecally produced;^87^ therefore, they may not necessarily be associated with plasma anti‐amyloid antibodies. However, naturally occurring plasma anti‐amyloid antibodies have been described in AD.^90^, ^91^ While several studies have demonstrated a reduction in the concentration of naturally occurring plasma anti‐amyloid antibodies in AD compared to HC,^90^, ^91^, ^92^, ^93^, ^94^ others have found elevations.^95^, ^96^ The clinical significance of these naturally occurring anti‐amyloid antibodies is uncertain, but they have been proposed to be protective,^97^ though in another study neither their presence nor concentration were associated with dementia risk.^98^ These naturally occurring amyloid antibodies in plasma may be related to cerebral amyloidosis, but no study to date has demonstrated an association with plasma antibodies and CAA‐ri.

## 5 DISCUSSION

Overall, this narrative review revealed several studies within the same participants directly comparing the diagnostic test performance of various plasma Aβ and tau assays in reference to PET or CSF reference standards for AD. We did not find any comparative studies with autopsy used as the diagnostic reference standard. Overall, both Simoa and IP‐MS methodologies showed good to excellent test performance for plasma tau biomarkers, while for plasma amyloid biomarkers the performance of Simoa was more variable and, in general, inferior to IP‐MS and LC‐MS.

Several studies to date demonstrate that the plasma Aβ_42/40_ ratio measured by IP‐MS could differentiate AD compared to reference standards of CSF Aβ_42/40_ and amyloid PET with an AUC ranging from 75% to 97%.^25^, ^28^, ^36^, ^99^, ^100^, ^101^ Though IP‐MS is more sensitive than immunoassays, one of the challenges is the need for technical expertise, larger sample volume, and, thus, it may be more difficult to scale up. Simoa is a high throughput, highly sensitive immune assay technology that is more accessible, but it does lack antibody specificity as some methods do not quantify the full length of Aβ and rather quantify amyloid fragments of various lengths.^10^ The first generation of Simoa Quanterix assays may not bind Aβ isoforms at the N‐terminus and, therefore, they can detect differing lengths of Aβ_x‐42_ and Aβ_x‐40_.^10^ The AUCs reported for Aβ_42/40_ for Simoa Quanterix assays have a much greater variability in the ability to differentiate AD groups based on CSF Aβ_42/40_, or amyloid PET, ranging from 0.58 to 0.92.^46^, ^102^, ^103^, ^104^, ^105^, ^106^, ^107^, ^108^, ^109^, ^110^, ^111^ For this reason IP‐MS methods, as evident in Table 1, are likely superior to Simoa methods for the quantification of Aβ in plasma.

Recognizing this limitation, novel modifications have attempted to increase Simoa's specificity. For instance the Simoa Amyblood method uses a more specific antibody quantifying the full length of Aβ_42_ and Aβ_40_.^35^, ^38^, ^43^ The results of our review do identify significant variability in the performance of Simoa assays for Aβ, suggesting the need for further optimization of Simoa technology for Aβ quantification specifically or potentially the use of IP‐MS or LC‐MS methods for Aβ quantification.

Another important consideration when evaluating different studies reporting variable AUCs is that differences in PET reference standards may have resulted in lower agreement of some Aβ_42/40_ assays compared to previously reported AUCs.^25^, ^28^ Florbetapir PET may have greater variability than PiB.^112^ However, an earlier study using the IP‐MS technique from Washington University used PiB‐PET and found that plasma Aβ_42/40_ had an AUC of 0.887,^113^ which is comparable to AUCs reported in Table 1 with the same IP‐MS technique from Washington University but using flutemetamol^34^ and florbetapir^35^ PET as reference standards. Furthermore, several studies have noted that the majority of discordant Aβ_42/40_ plasma and PET cases are Aβ_42/40_ plasma positive and amyloid‐PET negative.^28^, ^37^ Another important consideration when evaluating the test performance of methods in reference to PET is that it is possible that abnormal changes in plasma Aβ_42/40_ might precede the threshold at which Aβ cortical PET positivity is reached. This is consistent with prior work demonstrating that CSF Aβ changes are detected before Aβ PET positivity.^114^ Therefore, one important knowledge gap and direction for future studies is to evaluate how the diagnostic accuracy of plasma biomarkers may vary according to the stage of AD. Furthermore, there is a continuing need to evaluate potential sex differences in plasma biomarkers in AD and CAA.

While this narrative review focused on the comparisons of point estimate AUCs for individual biomarkers and different assays, when biomarkers are entered together in multivariable models, the sensitivity and specificity for discriminating the presence of AD can be improved. For instance, Meyer et al. found that IP‐MS Aβ_40/42_ and Simoa p‐tau231 resulted in a model with an AUC of 0.87 (95% CI: 0.80−0.94), while individually these biomarkers had AUCs of 0.81 (95% CI: 0.725−0.891) and 0.81 (95% CI: 0.71−0.91), respectively.^36^ In this study, p‐tau231 displayed greater association with tau PET and amyloid PET, compared to p‐tau181.^36^ Interestingly this study also noted that the ratio of the Aβ_40/42_ ratio to p‐tau231 predicted a faster decline in immediate and delayed memory domains in participants at risk of AD.^36^ In AD, neurofibrillary tangles are associated with the severity of AD dementia more than Aβ plaques,^115^ and it would be prudent to examine whether the same holds true in cases of CAA without AD. A combination of plasma p‐tau217, Aβ_42/40_, and NfL together with basic demographic data, predicted subsequent development of AD dementia with AUC = 0.82 (95% CI: 0.77–0.91) in an analysis of the BioFINDER study, thereby demonstrating the utility of plasma biomarkers evaluated combined with clinical factors to increase our diagnostic discriminative ability.^116^ Future studies examining the diagnostic capabilities of plasma biomarkers in CAA similarly should assess test performances individually for each biomarker, but also in combination in multivariable modeling.

In addition to the discriminative ability of plasma Aβ in discerning the presence of AD, plasma tau species have also emerged as valuable discriminative biomarkers. Among 1402 participants across three cohorts, p‐tau217 discriminated AD from other neurodegenerative diseases with higher accuracy than other plasma and neuroimaging biomarkers with an AUC 0.96 (95% CI: 0.93−0.98) using the Eli Lilly MSD immunoassay.^21^ In another study p‐tau181 and p‐tau231 measured via MSD immunoassay could discriminate AD from non‐AD neurodegenerative disease with AUCs > 0.90 and both were associated with amyloid and tau PET positivity.^117^ Furthermore, consistent across several different IP‐MS‐, MSD‐, and Simoa‐based technologies p‐tau217, p‐tau181, and p‐tau231 had good discriminative ability for predicting conversion from MCI to AD dementia and for discriminating AD based on CSF Aβ.^117^ The results in Table 2 and Figure 4, especially highlighting the study by Janelidze et al.,^42^ suggest that various Simoa, IP‐MS, and MSD assays had good to excellent performance for p‐tau isoforms.

This narrative review also identified a substantial knowledge gap in CAA. We did not find comparative studies evaluating the sensitivity and specificity of plasma biomarker assays in CAA. Furthermore, to date, no study has examined the sensitivity and specificity of plasma Aβ_40_, Aβ_42_, Aβ_40/42_, and p‐tau isoforms together in CAA.

The largest case–control study to date evaluating CSF biomarkers in CAA^61^ demonstrates largely consistent findings with prior work supporting that amyloid, particularly CSF Aβ_40_, is primarily reduced in CAA and that both CSF Aβ_40_ and Aβ_42_ have good to excellent diagnostic discriminative abilities.^61^ Overall, the consequences of parenchymal and vascular trapping of amyloid isoforms (depicted in Figure 2) are reductions in CSF Aβ_40_ and Aβ_42_.^61^, ^64^ The fact that Aβ_40_ was especially different between CAA and non‐CAA groups further supports the vascular propensity of Aβ_40_ and why it may be a hallmark biomarker in CAA.^61^, ^64^ Additionally, this study supports the hypothesis that elevations in t‐tau and p‐tau are greatest in AD, in which there is greater neuronal loss related to concomitant tauopathy, compared to CAA.^61^ Therefore, the combination of Aβ_40_, Aβ_42_, p‐tau, and t‐tau likely will help facilitate the differentiation between AD and CAA. Further, this combination may also help identify cases of concomitant CAA and AD. In these cases, it is possible that there will be elevations in tau isoforms that mirror those levels seen in AD and reductions in Aβ_40_ that mirror those levels seen in CAA. While insufficient evidence exists to conclude whether the same biomarker profile might occur in plasma, these findings would largely be supportive of the pathophysiologic model in Figure 2 and does invite future research to evaluate whether a potential plasma profile may be similar to the CSF profile identified.

There are limitations to the studies that have evaluated plasma or CSF biomarkers of CAA that are worth mentioning. First, most have done so in reference to possible and probable CAA and not definite, neuropathologically confirmed, CAA according to Boston criteria. Furthermore, across the studies evaluating CSF and plasma biomarkers of CAA, some only included those with probable CAA, while others included those with possible CAA, thereby adding to the heterogeneity in diagnostic certainty. Other studies also include those with acute ICH, adding further potential heterogeneity according to disease state or activity.^61^, ^68^, ^118^ It is uncertain whether plasma and/or CSF biomarkers may be altered in the setting of acute ICH. Studies evaluating plasma biomarkers in CAA have been especially limited by small sample sizes and variability in the methodologies used to quantify plasma biomarkers in CAA and, therefore, it is hard to draw definitive conclusions from this small body of literature. This calls for future studies in larger cohorts using robust plasma‐based methods to quantify and evaluate the core plasma biomarkers of CAA.

We have reached the point at which reliable tau and Aβ plasma assays have been developed in AD, and now efforts ought to examine the potential diagnostic utility and validation of these biomarkers and assays in CAA. Not only could plasma biomarkers increase the diagnostic sensitivity and specificity of the Boston criteria 2.0 for CAA even further, but there are clinical scenarios which may benefit from their implementation.

Having summarized several studies that directly compare different assays in the same patients with the same outcome measure (amyloid PET or CSF amyloid for instance) this review facilitates the identification of methods with the highest validity and reliability to quantify plasma biomarkers of AD in the future. The identified robust plasma methods in AD will help inform the selection of the best assays to evaluate potential biomarkers in CAA. Evaluating plasma biomarkers in CAA offers the potential to improve our clinical diagnostic approach. While there have been significant advances in the diagnosis and tailored treatments of acute ischemic stroke, both diagnostics and treatments for hemorrhagic stroke have lagged. In those with lobar hemorrhage, it can often be difficult to discern whether hemorrhage is due to CAA especially when neuroimaging biomarkers of CAA are not present.^2^, ^3^ Plasma biomarkers of CAA might improve our diagnostic accuracy in patients with ICH and could inform the future risk of hemorrhage. One study found that plasma full‐length Aβ_42_, truncated fragments, and full‐length Aβ_40_ were elevated in patients with CAA‐related ICH compared to HC.^71^ The clinical utility in measuring plasma biomarkers in CAA is also evidenced by another study, in which NfL predicted an increased risk of ICH.^119^ The validation of plasma biomarkers in CAA will also facilitate the evaluation of novel hypotheses. We postulate whether there may be a stage‐dependent alteration in plasma Aβ_40_ given its propensity to accumulate within cerebral small vessels in CAA. Some of the studies we identified, bearing in mind their small sample sizes, identified increases in plasma Aβ_40_ in CAA‐related hemorrhage.^72^ It is possible that during earlier stages of CAA plasma Aβ_40_ may be reduced in plasma, as it is in CSF, as at this stage Aβ_40_ is beginning to accumulate and damage cerebral small vessels. However, with progressive injury to cerebral small vessels it is not known whether plasma Aβ_40_ might change. Potentially, with increased spillover from microhemorrhage and lobar hemorrhage, there may be a rise in plasma Aβ_40_ (postulated in Figure 2). This hypothesis needs to be further evaluated as identifying such a rise in plasma Aβ_40_ could have wide implications for informing prognosis and the risk of hemorrhage in patients with CAA. Furthermore, the use of anticoagulation for other medical indications is not uncommon in elderly cohorts in which CAA can co‐occur. Plasma biomarkers of CAA, if validated and found to be predictive of hemorrhage, therefore, might also have potential implications to safely guide anticoagulation decision making as well in the future.

Evaluating potential plasma biomarkers of CAA also has relevance for the novel and emerging immunotherapies in AD, which facilitate the removal of cerebral Aβ.^82^ Therapies such as aducanumab^82^ and lecanemab^120^ for early AD facilitate Aβ clearance from the brain, but they can cause ARIA‐E and ARIA‐H, including microhemorrhage and leptomeningeal superficial siderosis.^82^ Those with apolipoprotein E (*APOEA*) ε4 positivity are at particular risk of ARIA.^82^ Amyloid‐targeting monoclonal antibodies, therefore, can induce an often subclinical neuroimaging pattern that resembles CAA and CAA‐ri.

Identifying a plasma biomarker profile of CAA‐ri could facilitate diagnosis of CAA‐ri and provide a more feasible, cost‐effective means of monitoring response to immunotherapy that could potentially guide the intensity of therapy. As CAA‐ri is rare, it would be prudent to support international collaboratives that store plasma samples from patients for future exploration of new markers. Future studies could use the most accurate and reliable plasma methods identified by this narrative review to explore potential differences in plasma Aβ_‐42/40_, t‐tau, p‐tau isoforms, GFAP, and NfL in those with CAA‐ri, CAA without inflammation, AD, and controls. One might hypothesize that, given the extent of neuroinflammation in CAA‐ri, a marker of reactive astrocytopathy, GFAP, may be particularly elevated in those with CAA‐ri. Additionally, studies are needed to determine whether plasma anti‐amyloid antibodies can be detected that correlate with the increased levels of CSF anti‐amyloid antibodies that are known to occur during CAA‐ri with active inflammation.

Overall, as immunotherapies for AD become increasingly used, there must be diagnostic prudence to identify those with concomitant CAA.^85^ Those with AD and concomitant CAA likely should not receive such therapies given their increased risk of hemorrhage.^83^, ^84^, ^85^ A plasma biomarker profile specific to CAA could facilitate the safe selection of patients with AD for disease‐modifying therapies. Furthermore, identifying plasma biomarkers of CAA might also help monitor the biological response to immunotherapies in AD, if vascular and parenchymal amyloid can be discriminated.^120^ The potential for plasma biomarkers to be used to monitor therapies is exhibited in the TRAILBLAZER‐ALZ randomized, double blind, placebo‐controlled clinical trial with donanemab, in which plasma levels of p‐tau217 and GFAP were significantly reduced in the donanemab treatment arm compared to placebo.^121^ In contrast to the development of therapeutics for AD, the therapeutic arena for CAA has been limited, perhaps out of concern that targeting Aβ in CAA might exacerbate the risk of hemorrhage. However, new apoE immunotherapies in animal models have been demonstrated to reduce amyloid pathology while preserving cerebrovascular integrity in CAA.^122^ To continue to advance the therapeutic front in CAA, we must also understand the unique plasma biomarker profile in CAA, whether this profile might predict risk of future hemorrhage, and how biomarkers might have a role in monitoring a biological response to emerging targeted therapies.

## 6 CONCLUSION

This narrative review provides a critical evaluation of the diagnostic test performances of several plasma Aβ_42_, Aβ_40_, and tau isoform assays in AD. Comparative studies consistently demonstrate that Simoa, MSD, and mass spectrometry methodologies have good to excellent test performance for plasma tau evaluation, while mass spectrometry methods consistently perform better for the quantification of plasma Aβ_42_ and Aβ_40_ isoforms. The summation and critique of knowledge presented in this review from such comparative studies and the summation of this body of knowledge into a schematic depiction of pathophysiologic underpinnings of CAA and the corresponding potential alterations in plasma biomarkers in Figure 2 is novel and will facilitate the selection of the most accurate and reliable methods for future research as well as the translation of these methods to investigate CAA.

Our review identifies a substantive knowledge gap in the assessment of potential plasma biomarkers in CAA and calls for dedicated research in this arena. We hypothesize that the identification of a novel plasma biomarker profile of CAA may additively facilitate its diagnosis in combination with Boston criteria and could, in the future, be predictive of prognosis and hemorrhage risk in CAA. The CSF biomarker profile of CAA is better characterized than plasma. The recently published, largest case–control study to date supports that CSF Aβ_42_ and Aβ_40_ isoforms have good to excellent discriminative ability for CAA.^61^ Reduced CSF t‐tau and p‐tau in CAA compared to AD may also be helpful.^61^, ^63^ Overall these studies invite expansion of research exploring potential plasma biomarkers in CAA. Aβ_40_ has a greater propensity for depositing in the small vessels of the cerebral vasculature and, hence, its greater specificity as a biomarker for CAA over AD in CSF studies to date. However, the limited literature examining plasma biomarkers in CAA have inconsistently identified both elevations and reductions in plasma Aβ_40_, highlighting an important need for further research using robust assays to clarify these discrepancies. We hypothesize that while changes in plasma Aβ_40_ levels might be congruous with CSF Aβ_40_, there could also be a stage‐dependent alteration in plasma Aβ_40_. Reductions in plasma Aβ_40_ may be congruous with reductions in CSF Aβ_40_ during earlier pre‐hemorrhagic stages of CAA, while elevations in plasma Aβ_40_ might occur in later hemorrhagic stages of CAA. This is a novel hypothesis that needs formal evaluation. Finally, our narrative review also places the utility of identifying a plasma biomarker profile of CAA in the context of emerging immunotherapies for AD. Given that immunotherapies, like lecanemab, can induce ARIA‐H and ARIA‐E and neuroimaging features that resemble CAA and CAA‐ri, identifying a sensitive and specific plasma biomarker profile of CAA could help prognosticate, monitor, and safely select those with AD for immunotherapies in the future.

## CONFLICT OF INTEREST STATEMENT

Dr. Ryan T. Muir does not report any actual or perceived conflicts of interest. Dr. Zahinoor Ismail reports consulting for Biogen, Roche, and Otsuka/Lundbeck, and is a site investigator for clinical trials sponsored by Avanir, ADDF, and NIA. Dr. Sandra E. Black reports: (1) grants or contracts: (1a) contract research (payments made to institution, no personal investigator fees taken): GE Healthcare, Genentech, Optina, Roche, Eli Lilly, Eisa/Biogen Idec, NovoNordisk, Lily Avid, (1b) peer review payments made to institution, no personal investigator fees taken): Ontario Brain Institute, CIHR, Leducq Foundation, Heart and Stroke Foundation of Canada, NIH, Alzheimer's Drug Discovery Foundation, Brain Canada, Weston Brain Institute, Canadian Partnership for Stroke Recovery, Canadian Foundation for Innovation, Focused Ultrasound Foundation, Alzheimer's Association US, Department of National Defence, Montreal Medical International Kuwait, Queen's University, Compute Canada Resources for Research Groups, CANARIE, Networks of Centres of Excellence of Canada; (2) consulting fees: Roche, Biogen, NovoNordisk; (3) payment or honoraria for lectures, presentations, speakers bureaus: Biogen; (4) monitoring board or advisory board (advisory boards only, no personal investigator fees taken): Conference Board of Canada, World Dementia Council University of Rochester Contribution to the Mission and Scientific Leadership of the Small Vessel VCID Biomarker Validation Consortium, National Institute of Neurological Disorders and Stroke. Dr. Eric E. Smith reports consulting for Eli Lilly and Alnylam and is a site investigator for a clinical trial sponsored by Biogen. Author disclosures are available in the supporting information.

## Supporting information

Supplementary Information

Supplementary Information
