# Supplementary material for: Comparative methods for quantifying plasma biomarkers in Alzheimer's disease: Implications for the next frontier in cerebral amyloid angiopathy diagnostics
Source: Alzheimers Dement. 2023 Oct 31;20(2):1436–58. doi: 10.1002/alz.13510 (PMC10916950; doi:10.1002/alz.13510)

Supplementary Table 1: Scale for the Assessment of Narrative Review Articles (SANRA)

Item	Author Statements
**Justification of the article's importance for the readership**	Plasma biomarkers are emerging as accessible diagnostic tests for Alzheimer's Disease dementia. With the advent of single molecule protein detection (Simoa) and immunoprecipitation mass spectrometry (IP-MS) assays, the ability to detect these biomarkers in plasma has improved. However, many assays exist with variable diagnostic test performances. This narrative review summarizes studies that directly compare the diagnostic test performance of various plasma biomarker techniques. This review will facilitate a critical overview of the various methodologies now available to help facilitate the identification of the biomarkers and methods with the best accuracy and reliability for future use.<br><br>Furthermore, while plasma biomarkers have been examined in AD, there is limited research done in Cerebral Amyloid Angiopathy. This narrative review will also summarize CSF and plasma biomarker profiles of CAA. Novel immunotherapies for AD may induce amyloid related imaging abnormalities (ARIA) and radiographic features which appear similar to CAA and CAA-related inflammation. Identifying a plasma CAA signature, therefore, may, in future, facilitate the safe selection of patients for emerging immunotherapies.
**Statement of concrete/specific aims or formulation of questions**	1. To provide a focused overview of recent studies which have directly compared the performance of different assays used to quantify plasma biomarkers in AD in reference to gold standard diagnostic tests.<br>2. To review CSF and plasma biomarker studies that have been published to date in CAA.<br>3. To discuss implications of these findings for the diagnosis and treatment of AD and CAA.
**Description of the literature review**	MEDLINE/PubMed search strategies for the primary and secondary objectives are described in the methods section.
**Referencing**	The introduction, results, and discussion sections pertaining to plasma and CSF biomarkers of AD and CAA are extensively referenced throughout.
**Scientific Reasoning**	The reasoning and evidence to support the finding that mass spectrometry methods may better quantify plasma Aβ than Simoa methods is discussed throughout the results and discussion sections. Reasoning to support both mass spectrometry and Simoa methods have good to excellent test performance for tau isoforms is discussed and thoroughly referenced throughout. Finally, the discussed reasoning for a need for future studies evaluating plasma biomarkers in CAA is also well referenced and identified and gaps are discussed in detail.
**Appropriate presentation of data**	Results pertaining to the study objectives were extracted from identified articles from the literature review and are presented in an organized and tabulated form (**Tables 1 – 3**) and **Figures 3 – 4**. Furthermore, we have generated **Figure 1** to summarize the main radiographic characteristics of CAA as defined by the Boston Criteria v2.0 as well as **Figure 2**, which is a schematic depiction of the pathophysiologic spectrum of AD and CAA and potential corresponding changes in plasma biomarkers across this spectrum.

Supplemental Figure 1: Inclusion flow diagram of studies directly comparing plasma biomarkers.

Supplementary Figure 1: Inclusion flow diagram of studies directly comparing plasma biomarkers.

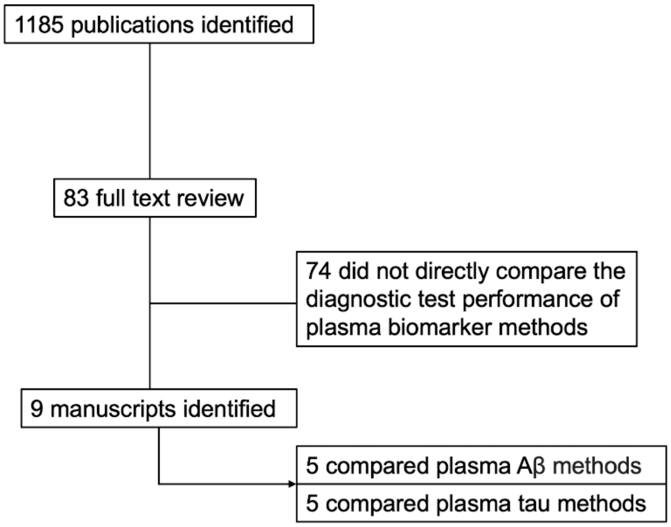

Supplement: Supplementary file 1 — Supplementary Information [file ALZ-20-1436-s001.pdf]
