# Supplementary material for: Comparative methods for quantifying plasma biomarkers in Alzheimer's disease: Implications for the next frontier in cerebral amyloid angiopathy diagnostics
Source: Alzheimers Dement. 2023 Oct 31;20(2):1436–58. doi: 10.1002/alz.13510 (PMC10916950; doi:10.1002/alz.13510)
Supplement: Supplementary file 2 — Supplementary Information [file ALZ-20-1436-s002.pdf]

ICMJE DISCLOSURE FORM

Date: 6/18/2023

Your Name: Zahinoor Ismail]

Manuscript Title: Comparative methods for quantifying plasma biomarkers in Alzheimer's disease: implications for the next frontier in cerebral amyloid angiopathy diagnostics

Manuscript Number (if known): Click or tap here to enter text.

In the interest of transparency, we ask you to disclose all relationships/activities/interests listed below that are related to the content of your manuscript. "Related" means any relation with for-profit or not-for-profit third parties whose interests may be affected by the content of the manuscript. Disclosure represents a commitment to transparency and does not necessarily indicate a bias. If you are in doubt about whether to list a relationship/activity/interest, it is preferable that you do so.

The author's relationships/activities/interests should be defined broadly. For example, if your manuscript pertains to the epidemiology of hypertension, you should declare all relationships with manufacturers of antihypertensive medication, even if that medication is not mentioned in the manuscript.

In item #1 below, report all support for the work reported in this manuscript without time limit. For all other items, the time frame for disclosure is the past 36 months.

		Name all entities with whom you have this relationship or indicate none (add rows as needed)	Specifications/Comments (e.g., if payments were made to you or to your institution)
**Time frame: Since the initial planning of the work**			
1	All support for the present manuscript (e.g., funding, provision of study materials, medical writing, article processing charges, etc.) **No time limit for this item.**	☒ None	
			Click the tab key to add additional rows.
**Time frame: past 36 months**			
**2**	Grants or contracts from any entity (if not indicated in item #1 above).	☐ **None**	
		NIA, CIHR, CCNA, Brain Canada, ADDF, Weston Foundation	
**3**	Royalties or licenses	☒ **None**	

		Name all entities with whom you have this relationship or indicate none (add rows as needed)	Specifications/Comments (e.g., if payments were made to you or to your institution)
**4**	Consulting fees	☒ **None**	
		Otsuka/Lundbeck	Paid to me
		Roche	Paid to institution
		Biogen	Paid to institution
**5**	Payment or honoraria for lectures, presentations, speakers bureaus, manuscript writing or educational events	☒ **None**	
		Otsuka/Lundbeck	Honoraria for presentations paid to me – unrelated to this work
**6**	Payment for expert testimony	☒ **None**	
**7**	Support for attending meetings and/or travel	☒ **None**	
**8**	Patents planned, issued or pending	☒ **None**	
**9**	Participation on a Data Safety Monitoring Board or Advisory Board	☐ **None**	
		OCEANS study Johns Hopkins DSMB	
**10**	Leadership or fiduciary role in other board, society, committee or advocacy group, paid or unpaid	☐ **None**	
		Chair Canadian Conference on Dementia	No conflict – voluntary position
		Chair Canadian Consensus Conference on Diagnosis and Treatment of Dementia	No conflict – voluntary position
		Scientific Advisory Council AAIC	No conflict – voluntary position

		Name all entities with whom you have this relationship or indicate none (add rows as needed)	Specifications/Comments (e.g., if payments were made to you or to your institution)
**11**	Stock or stock options	☒ **None**	
**12**	Receipt of equipment, materials, drugs, medical writing, gifts or other services	☒ **None**	
**13**	Other financial or non-financial interests	☒ **None**	

Please place an "X" next to the following statement to indicate your agreement:

☒ I certify that I have answered every question and have not altered the wording of any of the questions on this form.

ICMJE DISCLOSURE FORM

Date: 6/25/2023

Your Name: Ryan T. Muir

Manuscript Title: Comparative methods for quantifying plasma biomarkers in Alzheimer's disease: implications for the next frontier in cerebral amyloid angiopathy diagnostics

Manuscript Number (if known): Click or tap here to enter text.

In the interest of transparency, we ask you to disclose all relationships/activities/interests listed below that are related to the content of your manuscript. "Related" means any relation with for-profit or not-for-profit third parties whose interests may be affected by the content of the manuscript. Disclosure represents a commitment to transparency and does not necessarily indicate a bias. If you are in doubt about whether to list a relationship/activity/interest, it is preferable that you do so.

The author's relationships/activities/interests should be defined broadly. For example, if your manuscript pertains to the epidemiology of hypertension, you should declare all relationships with manufacturers of antihypertensive medication, even if that medication is not mentioned in the manuscript.

In item #1 below, report all support for the work reported in this manuscript without time limit. For all other items, the time frame for disclosure is the past 36 months.

		Name all entities with whom you have this relationship or indicate none (add rows as needed)	Specifications/Comments (e.g., if payments were made to you or to your institution)
		**Time frame: Since the initial planning of the work**	
1	All support for the present manuscript (e.g., funding, provision of study materials, medical writing, article processing charges, etc.) **No time limit for this item.**	☐ None	
		Canadian Institutes for Health Research – Graduate Student Award	Scholarship for graduate work
		Hotchkiss Brain Institute Denyse-Lajoie Lake Fellowship Award in Brain Research	Scholarship for fellowship research work
			Click the tab key to add additional rows.
		**Time frame: past 36 months**	
2	Grants or contracts from any entity (if not indicated in item #1 above).	☒ **None**	
3	Royalties or licenses	☒ **None**	

		Name all entities with whom you have this relationship or indicate none (add rows as needed)	Specifications/Comments (e.g., if payments were made to you or to your institution)
**4**	Consulting fees	☒ **None**	
**5**	Payment or honoraria for lectures, presentations, speakers bureaus, manuscript writing or educational events	☒ **None**	
**6**	Payment for expert testimony	☒ **None**	
**7**	Support for attending meetings and/or travel	☒ **None**	
**8**	Patents planned, issued or pending	☒ **None**	
**9**	Participation on a Data Safety Monitoring Board or Advisory Board	☒ **None**	
**10**	Leadership or fiduciary role in other board, society, committee or advocacy group, paid or unpaid	☐ **None**	
		Sunnybrook Health Sciences Research Ethics Board Member 2020 - 2023	

		Name all entities with whom you have this relationship or indicate none (add rows as needed)	Specifications/Comments (e.g., if payments were made to you or to your institution)
**11**	Stock or stock options	☒ **None**	
**12**	Receipt of equipment, materials, drugs, medical writing, gifts or other services	☒ **None**	
**13**	Other financial or non-financial interests	☒ **None**	

Please place an "X" next to the following statement to indicate your agreement:

☒ I certify that I have answered every question and have not altered the wording of any of the questions on this form.

ICMJE DISCLOSURE FORM

**Date:**	6/23/2023
**Your Name:**	Eric Smith
**Manuscript Title:**	Comparative methods for quantifying plasma biomarkers in Alzheimer's disease: implications for the next frontier in cerebral amyloid angiopathy diagnostics
**Manuscript Number (if known):**	Click or tap here to enter text.

In the interest of transparency, we ask you to disclose all relationships/activities/interests listed below that are related to the content of your manuscript. "Related" means any relation with for-profit or not-for-profit third parties whose interests may be affected by the content of the manuscript. Disclosure represents a commitment to transparency and does not necessarily indicate a bias. If you are in doubt about whether to list a relationship/activity/interest, it is preferable that you do so.

The author's relationships/activities/interests should be defined broadly. For example, if your manuscript pertains to the epidemiology of hypertension, you should declare all relationships with manufacturers of antihypertensive medication, even if that medication is not mentioned in the manuscript.

In item #1 below, report all support for the work reported in this manuscript without time limit. For all other items, the time frame for disclosure is the past 36 months.

		**Name all entities with whom you have this relationship or indicate none (add rows as needed)**	**Specifications/Comments (e.g., if payments were made to you or to your institution)**
		Time frame: Since the initial planning of the work	
1	All support for the present manuscript (e.g., funding, provision of study materials, medical writing, article processing charges, etc.) **No time limit for this item.**	☒ None	
			Click the tab key to add additional rows.
		Time frame: past 36 months	
**2**	Grants or contracts from any entity (if not indicated in item #1 above).	☐ **None**	
		Biogen	Payments made to the University of Calgary to participate as a site in a clinical trial.
**3**	Royalties or licenses	☒ **None**	

		Name all entities with whom you have this relationship or indicate none (add rows as needed)	Specifications/Comments (e.g., if payments were made to you or to your institution)
**4**	Consulting fees	☐ **None**	
		Eli Lilly	Personal consulting fees.
		Alnylam	Personal consulting fees.
**5**	Payment or honoraria for lectures, presentations, speakers bureaus, manuscript writing or educational events	☒ **None**	
**6**	Payment for expert testimony	☒ **None**	
**7**	Support for attending meetings and/or travel	☒ **None**	
**8**	Patents planned, issued or pending	☒ **None**	
**9**	Participation on a Data Safety Monitoring Board or Advisory Board	☒ **None**	
**10**	Leadership or fiduciary role in other board, society, committee or advocacy group, paid or unpaid	☒ **None**	

		Name all entities with whom you have this relationship or indicate none (add rows as needed)	Specifications/Comments (e.g., if payments were made to you or to your institution)
**11**	Stock or stock options	☒ **None**	
**12**	Receipt of equipment, materials, drugs, medical writing, gifts or other services	☒ **None**	
**13**	Other financial or non-financial interests	☒ **None**	

Please place an "X" next to the following statement to indicate your agreement:

☒ I certify that I have answered every question and have not altered the wording of any of the questions on this form.

ICMJE DISCLOSURE FORM

**Date:**	6/23/2023
**Your Name:**	Sandra E. Black
**Manuscript Title:**	Comparative methods for quantifying plasma biomarkers in Alzheimer's disease: implications for the next frontier in cerebral amyloid angiopathy diagnostics
**Manuscript Number (if known):**	Click or tap here to enter text.

In the interest of transparency, we ask you to disclose all relationships/activities/interests listed below that are related to the content of your manuscript. "Related" means any relation with for-profit or not-for-profit third parties whose interests may be affected by the content of the manuscript. Disclosure represents a commitment to transparency and does not necessarily indicate a bias. If you are in doubt about whether to list a relationship/activity/interest, it is preferable that you do so.

The author's relationships/activities/interests should be defined broadly. For example, if your manuscript pertains to the epidemiology of hypertension, you should declare all relationships with manufacturers of antihypertensive medication, even if that medication is not mentioned in the manuscript.

In item #1 below, report all support for the work reported in this manuscript without time limit. For all other items, the time frame for disclosure is the past 36 months.

		**Name all entities with whom you have this relationship or indicate none (add rows as needed)**	**Specifications/Comments (e.g., if payments were made to you or to your institution)**
**Time frame: Since the initial planning of the work**			
1	All support for the present manuscript (e.g., funding, provision of study materials, medical writing, article processing charges, etc.) **No time limit for this item.**	☒ None	
			Click the tab key to add additional rows.
**Time frame: past 36 months**			
**2**	Grants or contracts from any entity (if not indicated in item #1 above).	☐ **None**	
		Contract Research: GE Healthcare, Genentech, Optina, Roche, Eli Lilly, Eisa/Biogen Idec, NovoNordisk, Lilly Avid	Payments made to Institution. No personal investigator fees taken including Eli Lilly.
		Peer Reviewed: Ontario Brain Institute, CIHR, Leducq Foundation, Heart and Stroke Foundation of Canada, NIH, Alzheimer's Drug Discovery Foundation, Brain Canada, Weston Brain Institute, Canadian Partnership for Stroke Recovery, Canadian Foundation for Innovation, Focused Ultrasound Foundation, Alzheimer's Association US, Department of National Defence, Montreal Medical International Kuwait, Queen's University, Compute Canada Resources for Research Groups,	Payments made to Institution. No personal investigator fees taken.

		Name all entities with whom you have this relationship or indicate none (add rows as needed)	Specifications/Comments (e.g., if payments were made to you or to your institution)
		CANARIE, Networks of Centres of Excellence of Canada	
3	Royalties or licenses	☒ None	
4	Consulting fees	☐ None	
		Roche	Payments made to me
		Biogen	Payments made to me
		NovoNordisk	Payments made to me
5	Payment or honoraria for lectures, presentations, speakers bureaus, manuscript writing or educational events	☐ None	
		Biogen	Payments made to me
		Roche New England Journal Manuscript	No Payments to me
		Roche Models of Care Analysis in Canada in Submission	Payments made to me for advising and manuscript review
6	Payment for expert testimony	☒ None	
7	Support for attending meetings and/or travel	☒ None	
8	Patents planned, issued or pending	☒ None	
9	Participation on a Data Safety	☐ None	

		Name all entities with whom you have this relationship or indicate none (add rows as needed)	Specifications/Comments (e.g., if payments were made to you or to your institution)
	Monitoring Board or Advisory Board	Conference Board of Canada<br>World Dementia Council<br>University of Rochester Contribution to the Mission and Scientific Leadership of the Small Vessel VCID Biomarker Validation Consortium<br>National Institute of Neurological Disorders and Stroke<br>Eisai National Advisory Board on Lecanemab for Alzheimer's Disease	Advisory Boards only.<br>No personal fees taken.
10	Leadership or fiduciary role in other board, society, committee or advocacy group, paid or unpaid	☒ **None**	
11	Stock or stock options	☒ **None**	
12	Receipt of equipment, materials, drugs, medical writing, gifts or other services	☒ **None**	
13	Other financial or non-financial interests	☒ **None**	

Please place an "X" next to the following statement to indicate your agreement:

☒ I certify that I have answered every question and have not altered the wording of any of the questions on this form.
